# Supplementary material for: The RAS‐related GTPase RHOB confers resistance to EGFR‐tyrosine kinase inhibitors in non‐small‐cell lung cancer via an AKT‐dependent mechanism
Source: EMBO Mol Med. 2016 Dec 22;9(2):238–50. doi: 10.15252/emmm.201606646 (PMC5286377; doi:10.15252/emmm.201606646)
Supplement: Supplementary file 1 — Appendix [file EMMM-9-238-s001.pdf]

Appendix Table S1

	event / n	median PFS (month)	IC95%	p value
Age at diagnosis				p= 0.4284
≤ 65 years	43 / 47	8.6	[5.9 ; 13.4]	
> 65 years	37 / 48	13.1	[10.5 ; 15.3]	
Age at initiation of Tki treatment				p= 0.5227
≤ 65 years	43 / 47	9.1	[5.9 ; 13.6]	
> 65 years	38 / 49	13.1	[10.5 ; 15.3]	
Sex				p= 0.1953
Male	23 / 27	13.6	[6.4 ; 15.3]	
Female	58 / 69	11.9	[6.9 ; 13.7]	
Tobacco				p= 0.9798
no	52 / 60	12.1	[6.4 ; 14.4]	
yes	26 / 33	11.9	[8.1 ; 15.9]	
Type of EGFR-TKI				**p= 0.0134**
Gefitinib	46 / 51	10.3	[5.9 ; 12.4]	
Erlotinib (43) or Afatinib (2)	35 / 45	14.4	[8.1 ; 18.2]	
First-line EGFR-TKI treatment				p= 0.3204
No	26 / 33	11.8	[5.4 ; 18.2]	
Yes	55 / 63	12.3	[7.9 ; 14.1]	
Time between diagnosis and beginning of EGFR-TKI treatment				p= 0.1914
≤ 3 months	54 / 60	10.8	[6.4 ; 13.6]	
> 3 months	26 / 35	13.1	[6.4 ; 23.1]	
EGFR exon 19 deletion				p= 0.8943
No	28 / 35	12.4	[5.8 ; 15.3]	
Yes	53 / 61	12.1	[8.1 ; 14.4]	
EGFR exon 21 mutation				p= 0.9009
No	54 / 62	11.9	[7.9 ; 14.4]	
Yes	23 / 30	12.4	[5.8 ; 15.3]	
RHOB staining				**p< 0.0001**
0 / +	46 / 57	15.3	[13.1 ; 18.2]	
++ / +++	35 / 39	5.6	[3.6 ; 6.4]	

Appendix Table S2

	HR [IC95%]	p value
Type of EGFR-TKI		
Erlotinib or Afatinib	1.00	
Gefitinib	1.49 [0.94 ; 2.38]	p= 0.089
RHOB staining		
0 / +	1.00	
++ / +++	4.26 [2.56 ; 7.08]	**p< 0.001**

Appendix Table S3

		RHOB expression determined by IHC			RHOB expression determined by RT-qPCR *(n=20)*	
		0/+ *(n=7)*	++/+++ *(n=13)*	*p value*	correlation coefficient	*p value*
**miR-19a**	Median *(range)*	1.00 *(0.13 : 3.74)*	0.45 *(0.10 : 2.23)*	*0.1655*	-0.3248	*0.1623*
**miR-19b**	Median *(range)*	2.23 *(0.17 : 5.14)*	0.88 *(0.18 : 3.73)*	*0.4054*	-0.2241	*0.3423*
**miR-21**	Median *(range)*	1.01 *(0.57 : 3.70)*	0.36 *(0.09 : 2.46)*	***0.0079***	-0.6150	***0.0039***
**miR-30b**	Median *(range)*	1.00 *(0.50 : 8.55)*	1.80 *(0.36 : 15.49)*	*0.5523*	0.2872	*0.2195*
**miR-30e**	Median *(range)*	3.49 *(1.00 : 8.23)*	2.66 *(1.48 : 14.96)*	*0.6630*	0.0647	*0.7865*
**miR-223**	Median *(range)*	1.00 *(0.43 : 6.80)*	1.31 *(0.25 : 19.63)*	*0.5006*	0.2271	*0.3357*

Appendix Figure S1

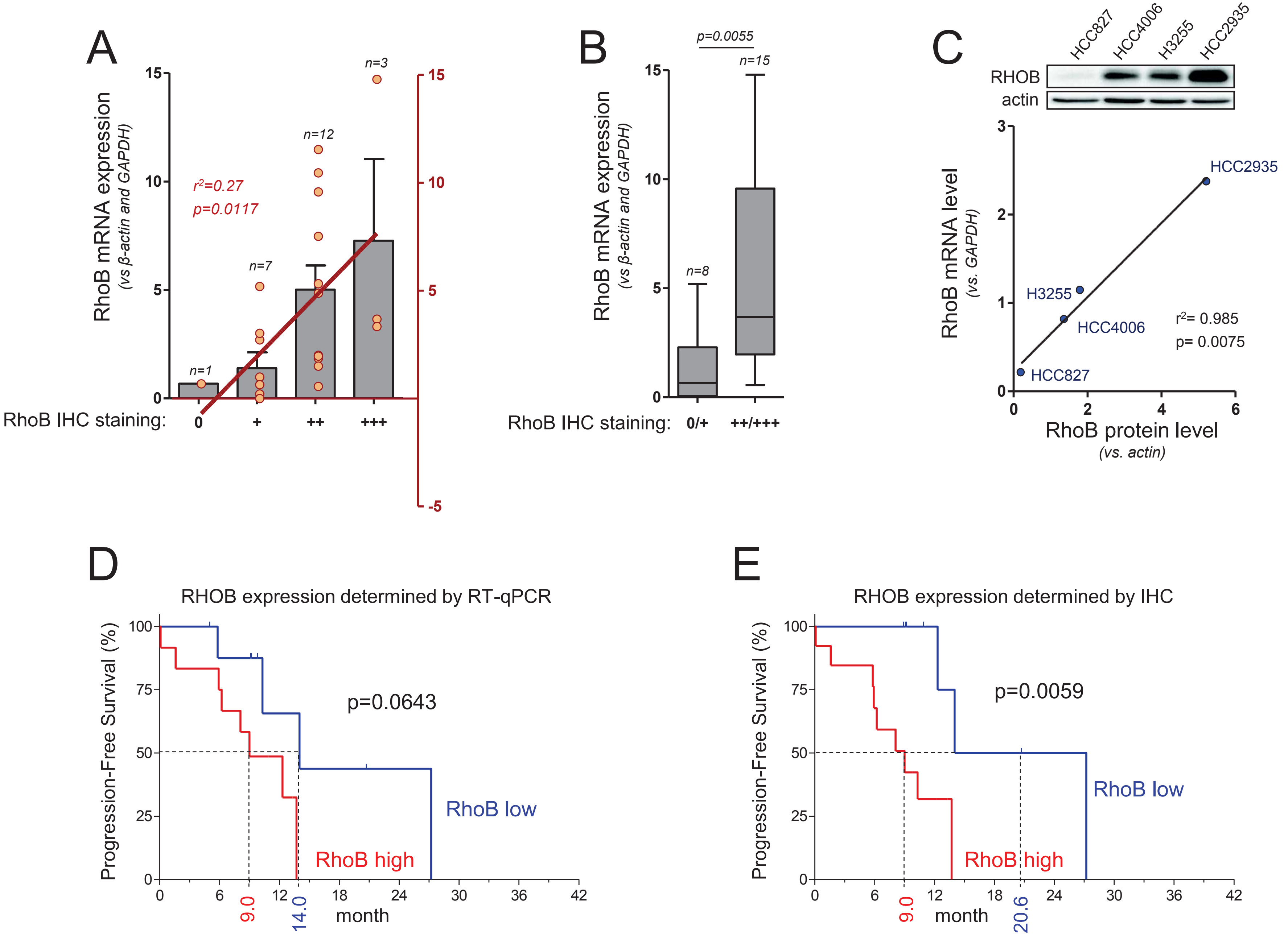

Appendix Figure S2

A

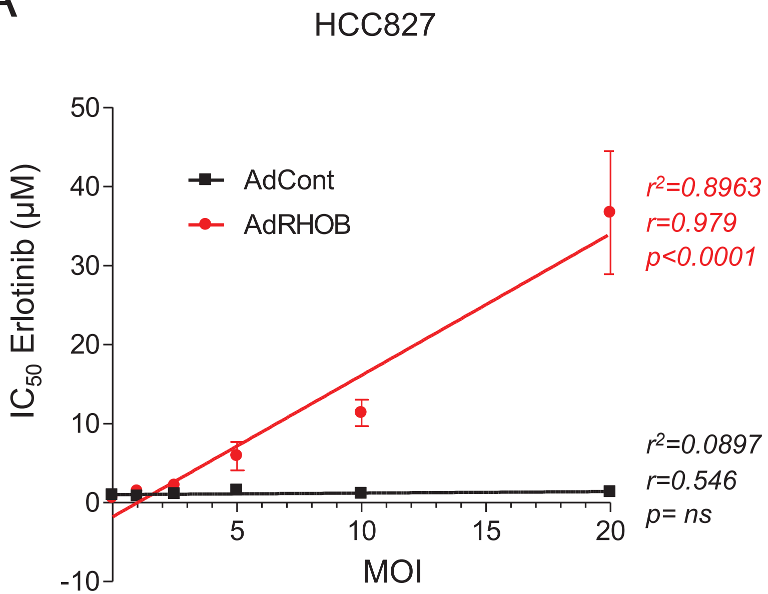

B

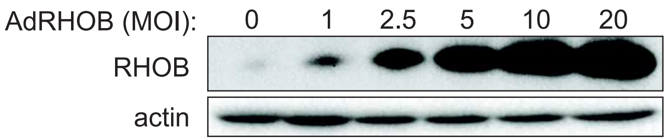

Appendix Figure S3

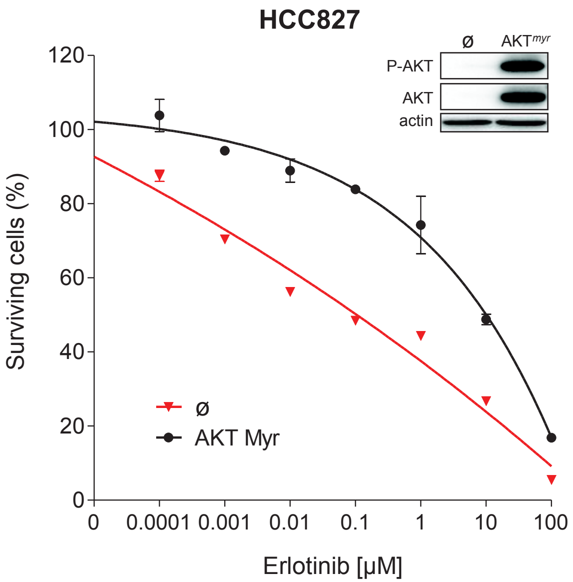

Appendix Figure S4

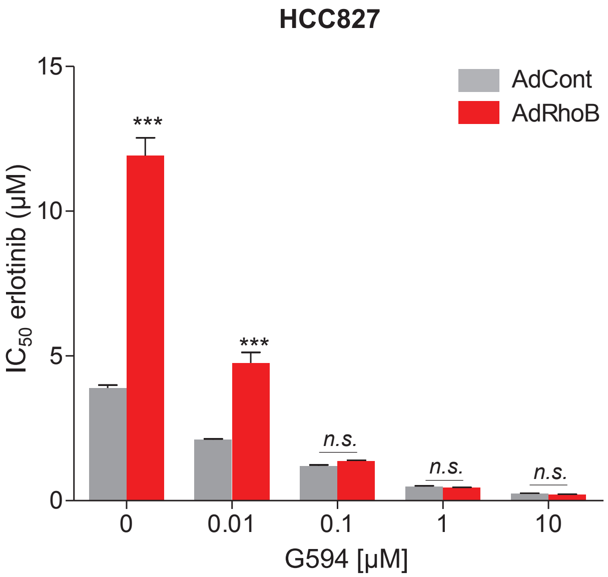

Appendix Figure S5

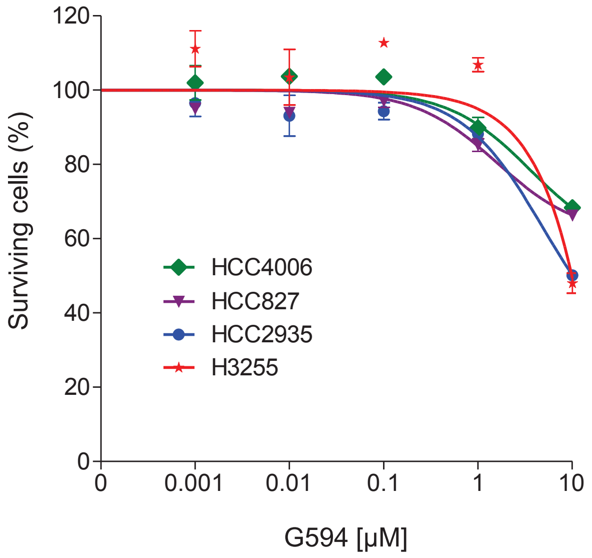

Appendix Figure S6

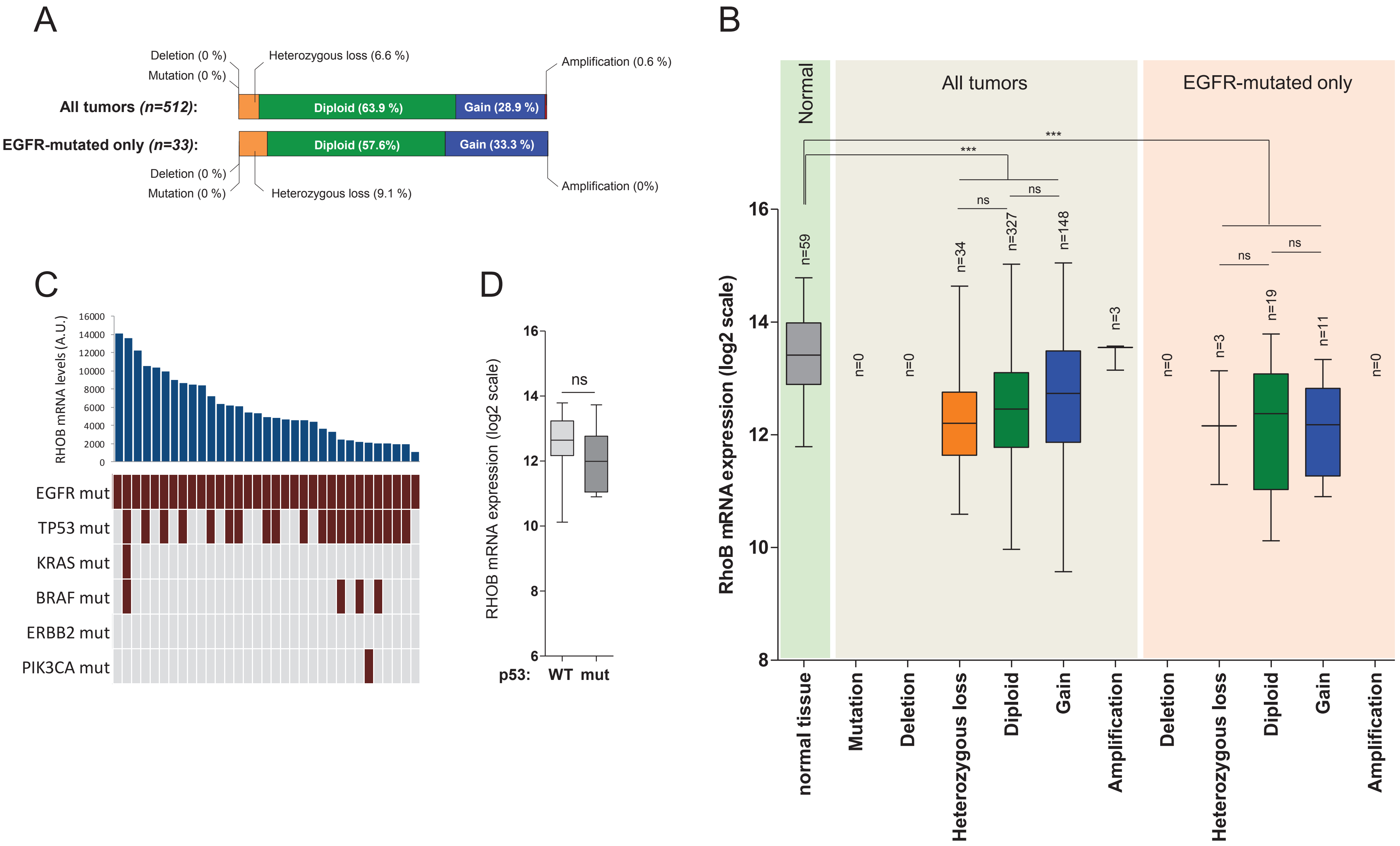

Appendix Figure S7

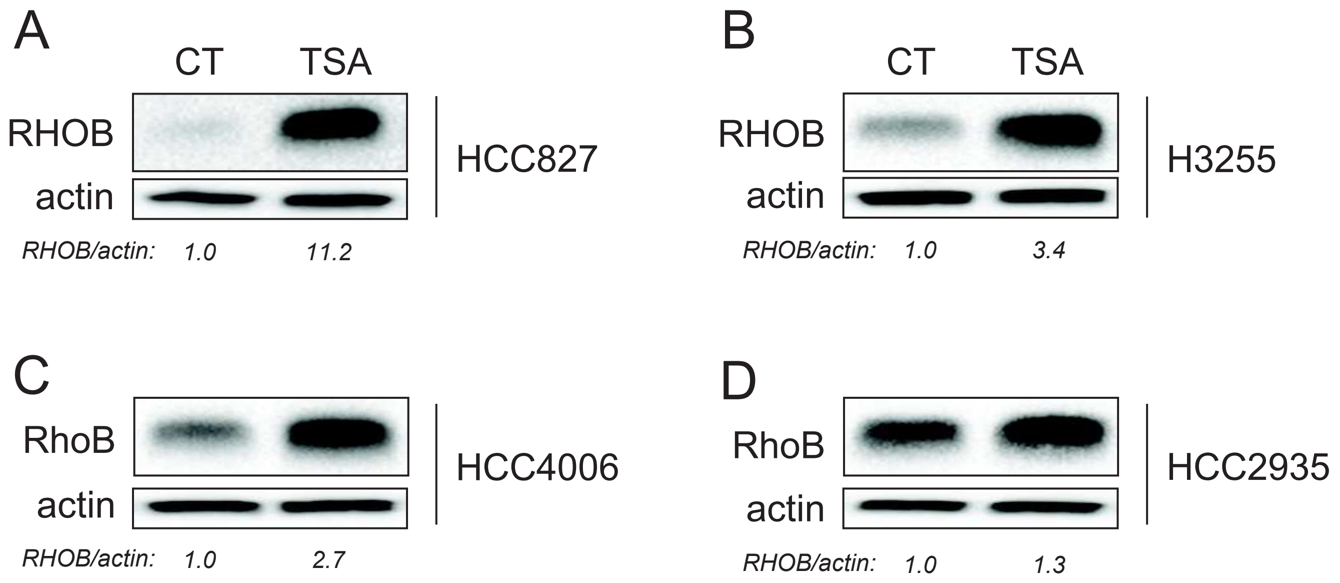

Legends to Supplementary Figures and Tables

Appendix Table S1. Univariate analysis of the 96 lung cancer patients harboring an EGFR-activating mutation. Univariate analysis of the 96 patients enrolled in the study. All patients had a grade IIIB (4 patients) or IV (92 patients) lung adenocarcinoma with an activating mutation in the EGFR gene, and were treated with an EGFR-TKI, either gefitinib (n=51), erlotinib (n=43) or afatinib (n=2). Progression-Free Survival (PFS) was determined by the Kaplan-Meier method.

Appendix Table S2. Multivariate analysis of the 96 lung cancer patients harboring an EGFR-activating mutation.

Appendix Table S3. Correlation between expression of several miRNA and RHOB levels determined by RT-qPCR or IHC staining in tumors of patients harboring EGFR-activating mutations.

Appendix Figure S1. RHOB mRNA and protein expression are correlated and are predictive of response to EGFR-TKI in EGFR-mutated patients. A) RHOB mRNA levels in tumors according to the corresponding IHC score (null: 0, weak: +, moderate: ++ and high: +++), including correlation analysis (in red, right Y axis). **B**) RHOB mRNA levels in the RHOB-low (0/+) and the RHOB-high (++/+++) groups. **C**) Correlation between RHOB mRNA and protein levels in four EGFR-mutated cell lines, determined by RT-qPCR and Western Blot, respectively. Progression-free survival of EGFR-TKI-treated patients with EGFR-mutated lung tumors, according to RHOB expression assessed by (**D**) quantitative real-time PCR (low RHOB group= under median, blue curve; high RHOB group = above median, red curve) for the subset of 21 patients, or by (**E**) IHC in the corresponding samples (low RHOB group= 0/+ staining; high RHOB group = ++/+++ staining).

Appendix Figure S2. RHOB expression correlates with erlotinib resistance *in vitro*. HCC827 cells were transduced with control (AdCont) or RHOB-overexpressing adenoviruses (AdRHOB) at an increasing multiplicity of infection (MOI). Erlotinib IC_{50} values were determined after 72 h by an MTS assay, then correlation analysis was performed (**A**). RHOB overexpression was monitored by western blotting (**B**).

Appendix Figure S3. AKT activation induces resistance to erlotinib in EGFR-mutated cell lines. HCC827 cells were transfected with a plasmid coding for a constitutively active AKT mutant (AKT^{myr}, *myristoylated*) or an empty vector (ø), then treated for 72 h with increasing concentrations of erlotinib. The surviving cell fraction was determined by an MTS assay. AKT overexpression and phosphorylation at Ser473 was assessed by western blotting.

Appendix Figure S4. The AKT inhibitor G594 reverses RHOB-induced erlotinib resistance in a dose-dependent manner. HCC827 cells were transduced with control (AdCont) or RHOB-overexpressing adenoviruses (AdRHOB) and treated for 72 h with increasing concentrations of erlotinib in the absence or presence of increasing doses of the AKT inhibitor G594. The surviving cell fraction was determined by an MTS assay, and the IC_{50} values were determined for each condition. (***: $p<0.0001$ *vs.* AdCont cells).

Appendix Figure S5. Effect of the AKT inhibitor G594 on cell survival. HCC4006, H827, HCC2935 and H3255 cells were treated for 72 h with increasing concentrations of the AKT inhibitor G594 and the surviving cell fraction was determined by an MTS assay.

Supplementary Figure S6. RHOB mRNA levels are not altered by copy number variations or major oncogenic mutations. All data were retrieved from the lung adenocarcinoma TCGA database, using the cBioPortal for Cancer Genomics tool (cbioportal.org). **A**) Percentage of RHOB mutation and putative copy number alterations in tumor samples from 512 lung adenocarcinoma patients, or in selected patients harboring an EGFR-mutation (n=33). Putative copy number was determined using GISTIC 2.0 (values: -2: homozygous deletion, -1: heterozygous loss, 0: diploid, +1: Gain, +2: amplification). **B**) Correlation between RHOB mRNA expression (Y axis, log2 scale) and copy number (X axis). **C**) Correlation between RHOB expression and the major oncogenic mutations found in EGFR-mutated lung adenocarcinomas. **D**) Expression of RHOB mRNA in p53 WT and p53 mutated lung adenocarcinomas.

Supplementary Figure S7. HDAC inhibition induces RHOB overexpression in EGFR-mutated cell lines. HCC827 (**A**), H3255 (**B**), HCC4006 (**C**) and HCC2935 (**D**) cell lines were treated for 24h with Trichostatin A (TSA) at 1 µM and RHOB expression was assessed by Western Blot and normalized by actin.

Supplementary Material

Quantitative real-time reverse transcription PCR

For RNA isolation, tumor areas (predetermined by an anatomopathologist) from 4-to-6 freshly cut sections (n=29) of FFPE tissue (10 µm thick) were collected using a scalpel, RNA extraction was performed using the miRNeasy FFPE (Qiagen) that allow extraction of total RNA including miRNA, following manufacturer's instructions. After extraction, 4 out of the 29 samples (13.8%) didn't show quantifiable amount of RNA (determined by NanoDrop® ND-1000 Spectrophotometer). For the remaining 25 samples, 100 ng of total RNA was reverse-transcribed using the iScript cDNA synthesis kit, following the manufacturer's instructions (Bio-Rad Laboratories), and quantitative real-time PCR of RhoB, β-actin and GAPDH mRNA was performed with a CFX96 detection system (Bio-Rad), using iQ SYBR Green Supermix (Bio-Rad) and the sequence-specific primers for RhoB (forward, 5-TTGTGCCTGTCCTAGAAGTG-3′; reverse, 5- CAAGTGTGGTCAGAATGCTAC-3′), β-actin (forward, 5-TCCCTGGAGAAGAGCTACGA-3′; reverse, 5-AGGAAGGAAGGCTGGAAGAG-3′) and GAPDH (forward, 5- TGCACCACCAACTGCTTAGC -3′; reverse, 5- GGCATGGACTGTGGTCATGAG-3′). The relative RhoB mRNA expression was calculated according to the $2^{-\Delta\Delta Cq}$ method, normalized to the β-actin and GAPDH mRNA levels. Two samples showed very late or no amplification of any of the three genes, probably due to a high degree of RNA degradation, and were excluded from the analysis. From the 23 RNA samples used for RHOB mRNA quantification, RNA was still available for 20 samples. 100 ng of total RNA (including miRNA) was reverse-transcribed using the Universal cDNA Synthesis Kit II, following the manufacturer's instructions (Exiqon), and quantitative real-time PCR of miR-19a, miR-19b, miR-21, miR-30b, miR-30e and miR-223 was performed with a CFX96 detection system (Bio-Rad), using miRCURY LNA microRNA PCR ExiLENT SYBR® Green (Exiqon) and the LNA™ PCR primer set for hsa-miR-19a-3p (#205862), hsa-miR-19b-3p (#204450), hsa-miR-21 (#204230), hsa-miR-30b-5p (#204765), hsa-miR-30e-3p (#204410), hsa-miR-223-3p (#204256), and reference primer mix for SNORD38B (#203901) and SNORD49A (#203904) for normalization.

TCGA data analysis

RHOB mRNA levels, putative copy number alteration (from GISTIC 2.0; values: -2: homozygous deletion, -1: heterozygous loss, 0: diploid, +1: Gain, +2: amplification), and mutational status were retrieved from the lung adenocarcinoma TCGA database (http://cancergenome.nih.gov/), using the cBioPortal for Cancer Genomics tool (cbioportal.org). Data were available for 512 lung adenocarcinoma samples and RHOB mRNA levels were available from 59 normal adjacent lung tissue samples.
