# Supplementary material for: The RAS‐related GTPase RHOB confers resistance to EGFR‐tyrosine kinase inhibitors in non‐small‐cell lung cancer via an AKT‐dependent mechanism
Source: EMBO Mol Med. 2016 Dec 22;9(2):238–50. doi: 10.15252/emmm.201606646 (PMC5286377; doi:10.15252/emmm.201606646)
Supplement: Supplementary file 2 — Expanded View Figures PDF [file EMMM-9-238-s002.pdf]

Expanded View Figures

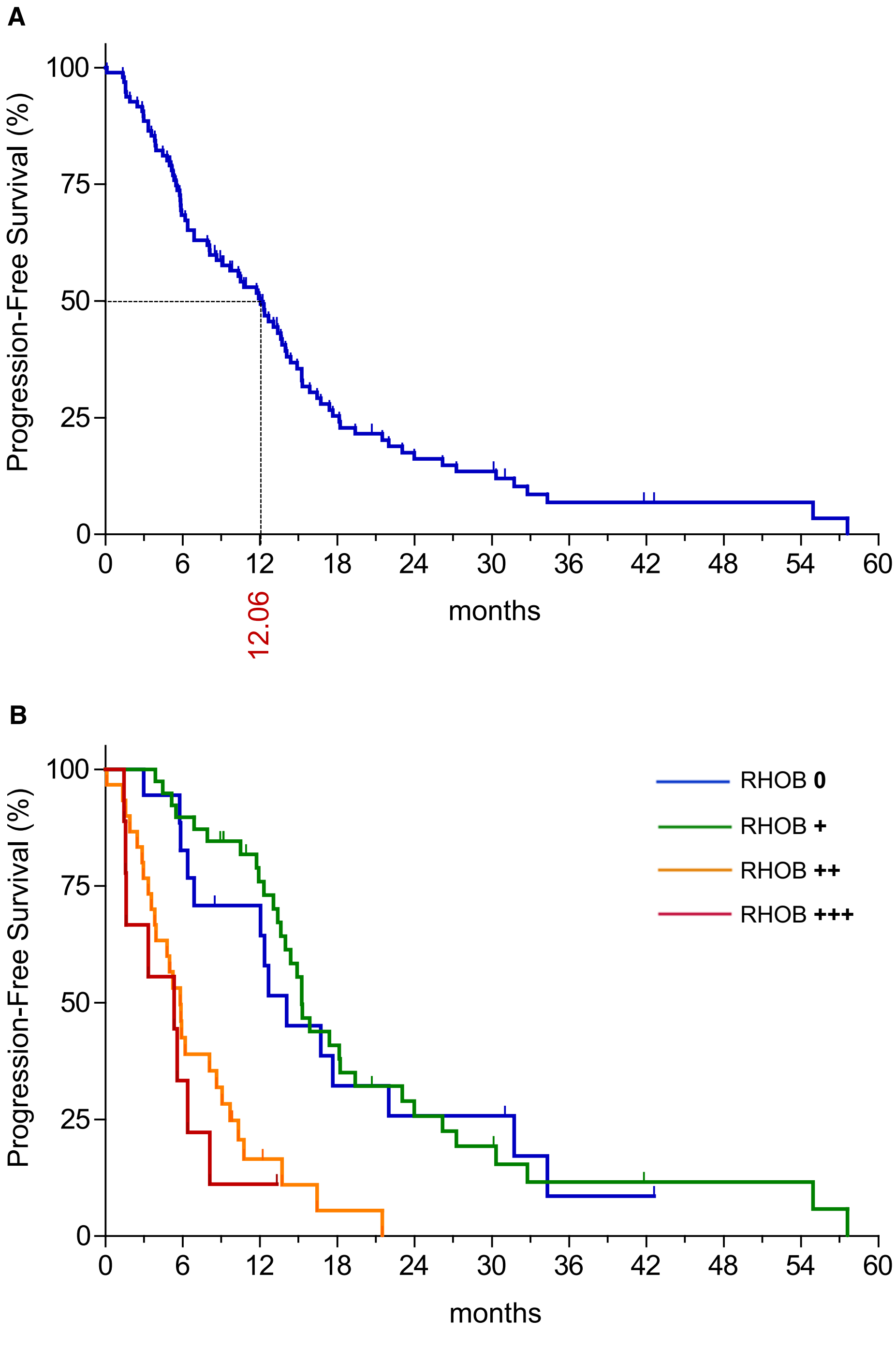

Figure EV1. RHOB expression predicts the response to EGFR-TKI in patients with EGFR-mutated lung cancer.

A, B Progression-free survival of EGFR-mutated patients treated with EGFR-TKI (A) for the whole population or (B) according to RHOB expression in treatment-naive tumors (null: 0; weak: +; moderate: ++; and high: +++), as assessed by immunohistochemistry.

© 2016 The Authors

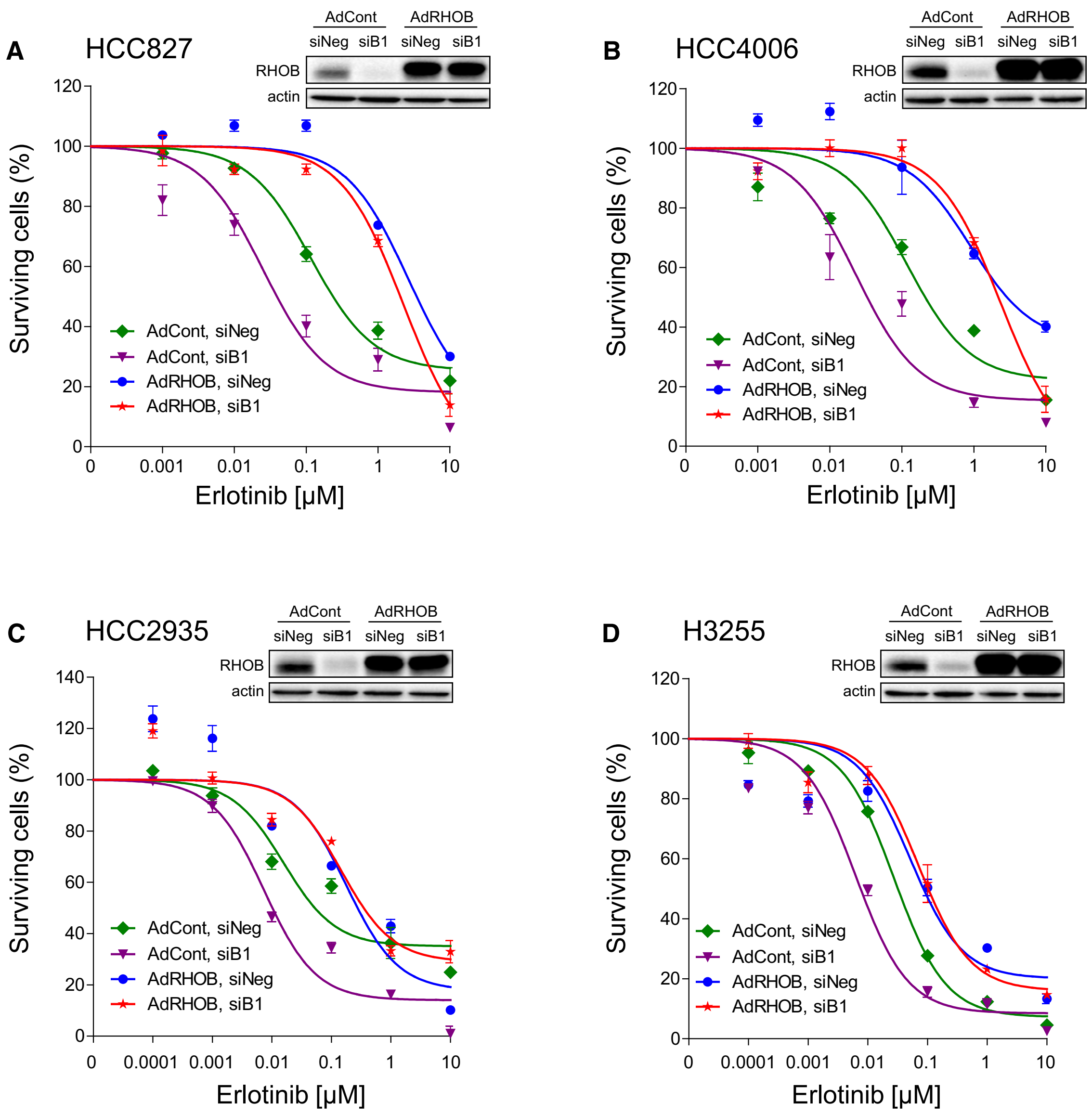

Figure EV2. Adenoviral-mediated RHOB overexpression reverses erlotinib sensitivity induced by RHOB depletion.

A–D HCC827 (A), HCC4006 (B), H3255 (C), or HCC2935 (D) cells were transfected with control (siNeg) or RHOB-targeting siRNA (siB1) and then transduced with control (AdCont) or RHOB-overexpressing (AdRHOB) adenoviruses and treated with increasing doses of erlotinib. The surviving cell fraction was determined by an MTS assay after 72 h and compared to untreated cells. RHOB overexpression or inhibition was monitored by Western blotting. Data are expressed as mean ± SEM from three independent experiments.

Source data are available online for this figure.

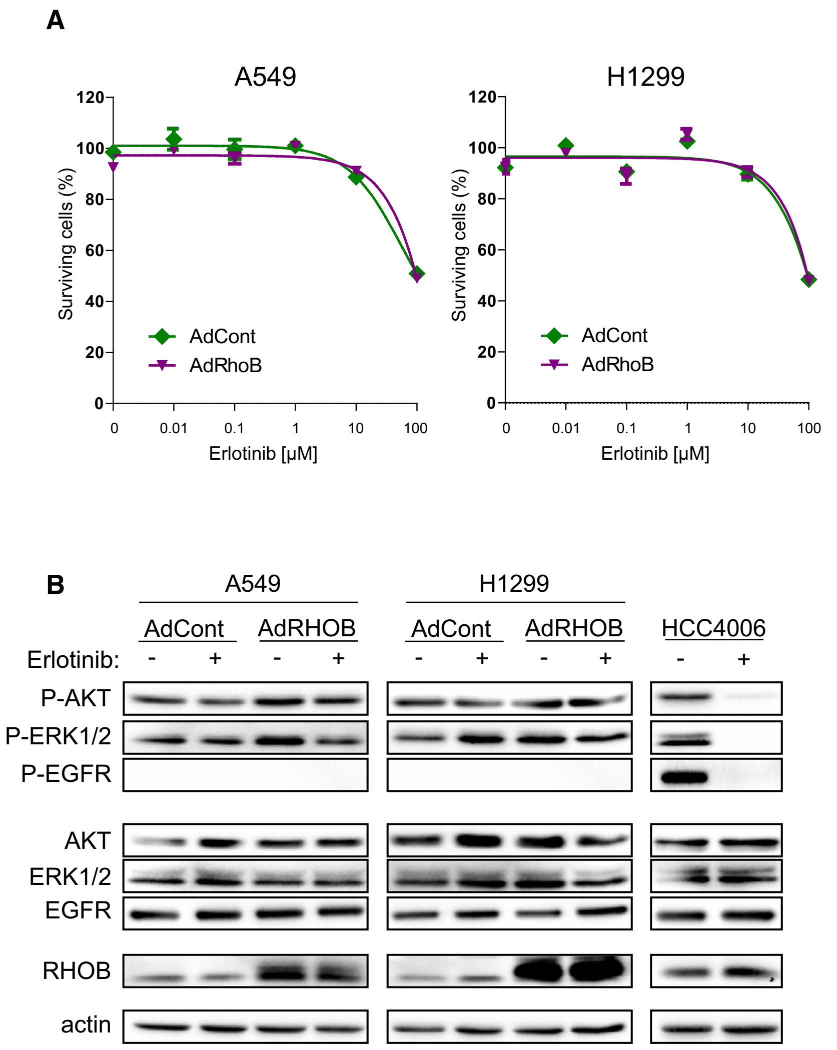

Figure EV3. RHOB overexpression does not affect response to erlotinib in EGFR WT cell lines.

A A549 or H1299 cells were transduced with control (AdCont) or RHOB-overexpressing adenoviruses (AdRHOB) and treated with increasing doses of erlotinib. The surviving cell fraction was determined by an MTS assay after 72 h and compared to untreated cells. Data are expressed as mean ± SEM from three independent experiments.

B A549 and H1299 cells were transduced with control (AdCont) or RHOB-overexpressing (AdRHOB) adenoviruses and treated for 4 h with erlotinib at 1 µM. The phosphorylation status of AKT, ERK1/2, and EGFR was assessed by Western blotting and normalized according to total protein levels. RHOB overexpression was also monitored by Western blotting. EGFR-mutated HCC4006 cells were used to monitor erlotinib efficiency (right panel).

Source data are available online for this figure.

Figure EV4. G594 prevents GSK3β phosphorylation in RHOB-overexpressing cells treated with erlotinib and reverses RHOB-induced resistance.

A–C H3255 (A), HCC2935 (B), and HCC827 (C) cells were transduced with control (AdCont) or RHOB-overexpressing (AdRHOB) adenoviruses and treated for 4 h with erlotinib (100 nM), G594 (100 nM), or a combination of both drugs. The phosphorylation status of GSK3β (Ser9), ERK1/2, and EGFR (Tyr1173) was assessed by Western blotting and normalized according to the total protein levels. RHOB overexpression was also monitored by Western blotting.

D–F H3255 (D), HCC2935 (E), or HCC827 (F) cells were transduced with control (AdCont) or RHOB-overexpressing (AdRHOB) adenoviruses and treated for 72 h with erlotinib alone (black and red curves) or in combination with the AKT inhibitor G594 at 100 nM (green and blue curves). The surviving cell fraction was determined by an MTS assay. Data are expressed as mean ± SEM from three independent experiments.

Source data are available online for this figure.

 © 2016 The Authors

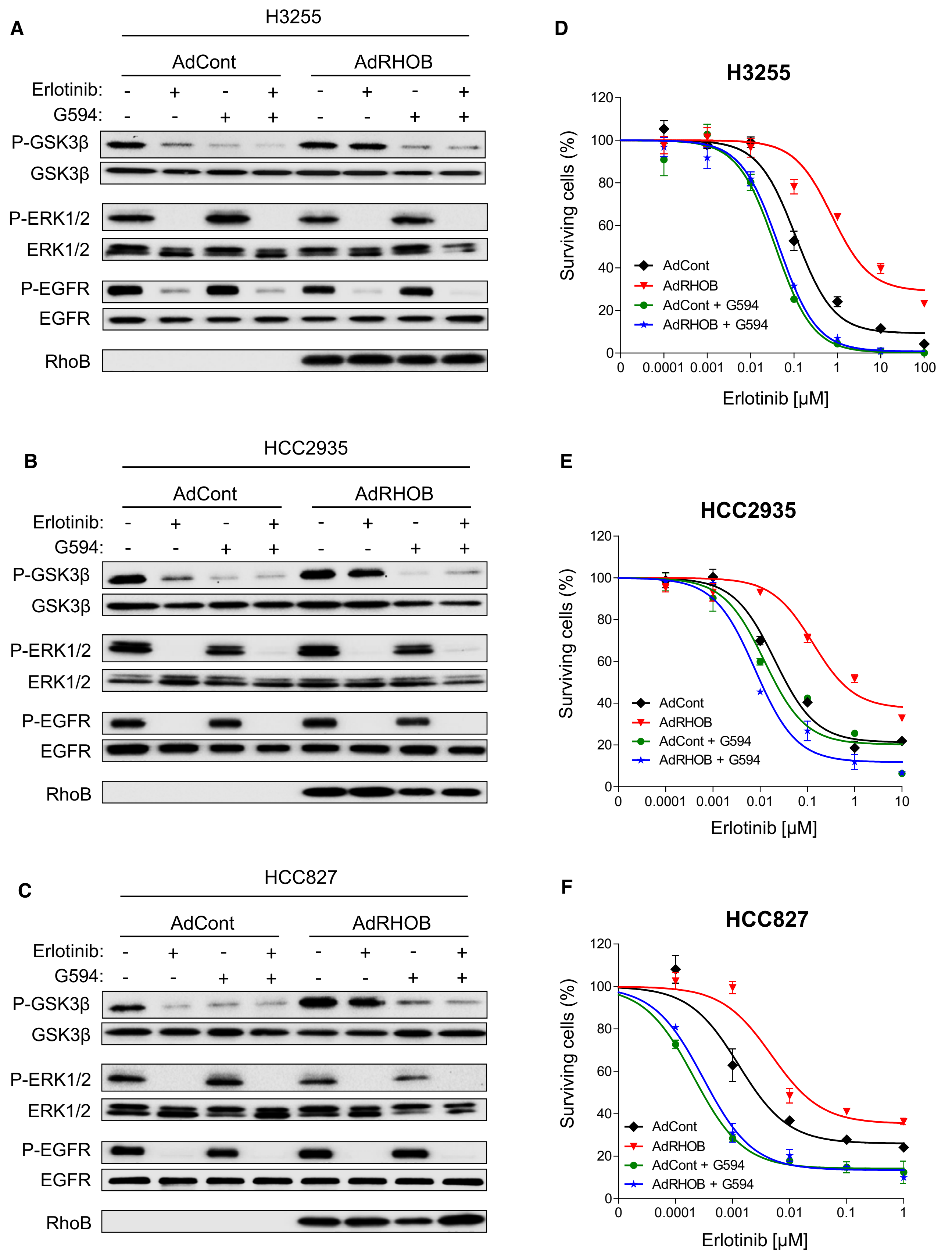
A
H3255
AdCont
AdRHOB
Erlotinib: - + - + - + - +
G594: - - + + - - + +
P-GSK3β
GSK3β
P-ERK1/2
ERK1/2
P-EGFR
EGFR
RhoB
D
H3255
Surviving cells (%)
Erlotinib [μM]
AdCont
AdRHOB
AdCont + G594
AdRHOB + G594
B
HCC2935
E
HCC2935
C
HCC827
F
HCC827

Figure EV4.

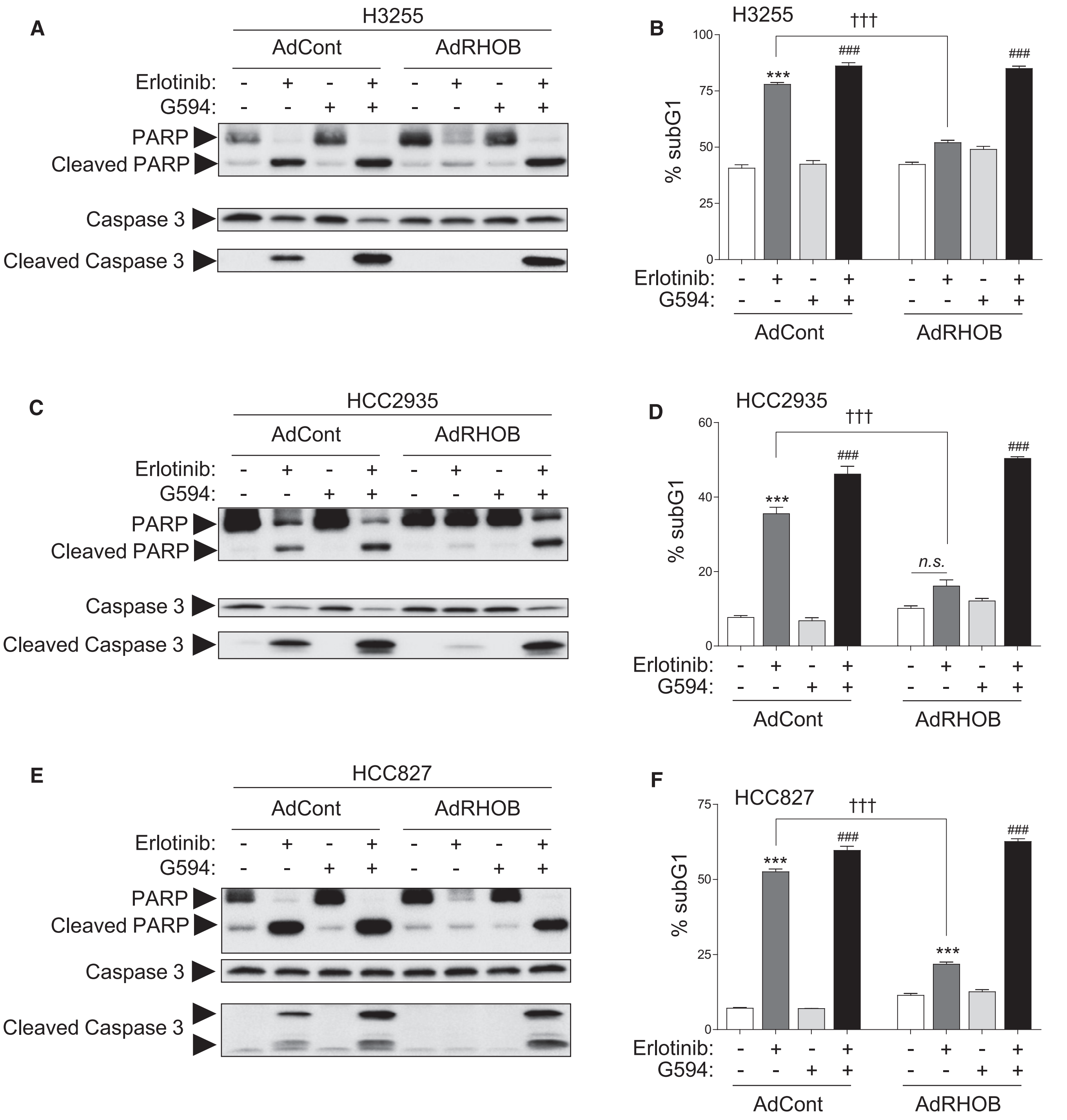

Figure EV5. AKT inhibition reverses RHOB-induced resistance to erlotinib-mediated apoptosis.

A–F H3255 (A and B), HCC2935 (C and D), or HCC827 (E and F) cells were transduced with control (AdCont) or RHOB-overexpressing (AdRHOB) adenoviruses and treated for 48 h with erlotinib (100 nM), G594 (100 nM), or a combination of both drugs. Apoptosis was then determined by either detection of cleaved PARP and caspase-3 (A, C, and E) or quantification of the subG1 cell population (B, D, and F). ***$P < 0.0001$ versus untreated cells; ###$P < 0.0001$ versus G594 treated cells; †††$P < 0.0001$ versus erlotinib-treated AdCont cells. Data are expressed as mean ± SEM from three independent experiments, *P*-values were determined by unpaired two-tailed Student's *t*-test.

Source data are available online for this figure.

 © 2016 The Authors
