# Supplementary material for: The RAS‐related GTPase RHOB confers resistance to EGFR‐tyrosine kinase inhibitors in non‐small‐cell lung cancer via an AKT‐dependent mechanism
Source: EMBO Mol Med. 2016 Dec 22;9(2):238–50. doi: 10.15252/emmm.201606646 (PMC5286377; doi:10.15252/emmm.201606646)
Supplement: Supplementary file 3 — Source Data for Expanded View and Appendix [file EMMM-9-238-s006.zip › EMM_06646_EV_Appendix_Source_Data/Figure_EV3/EMM_06646_Fig_EV3_source_data.pdf]

Fig. EV3B

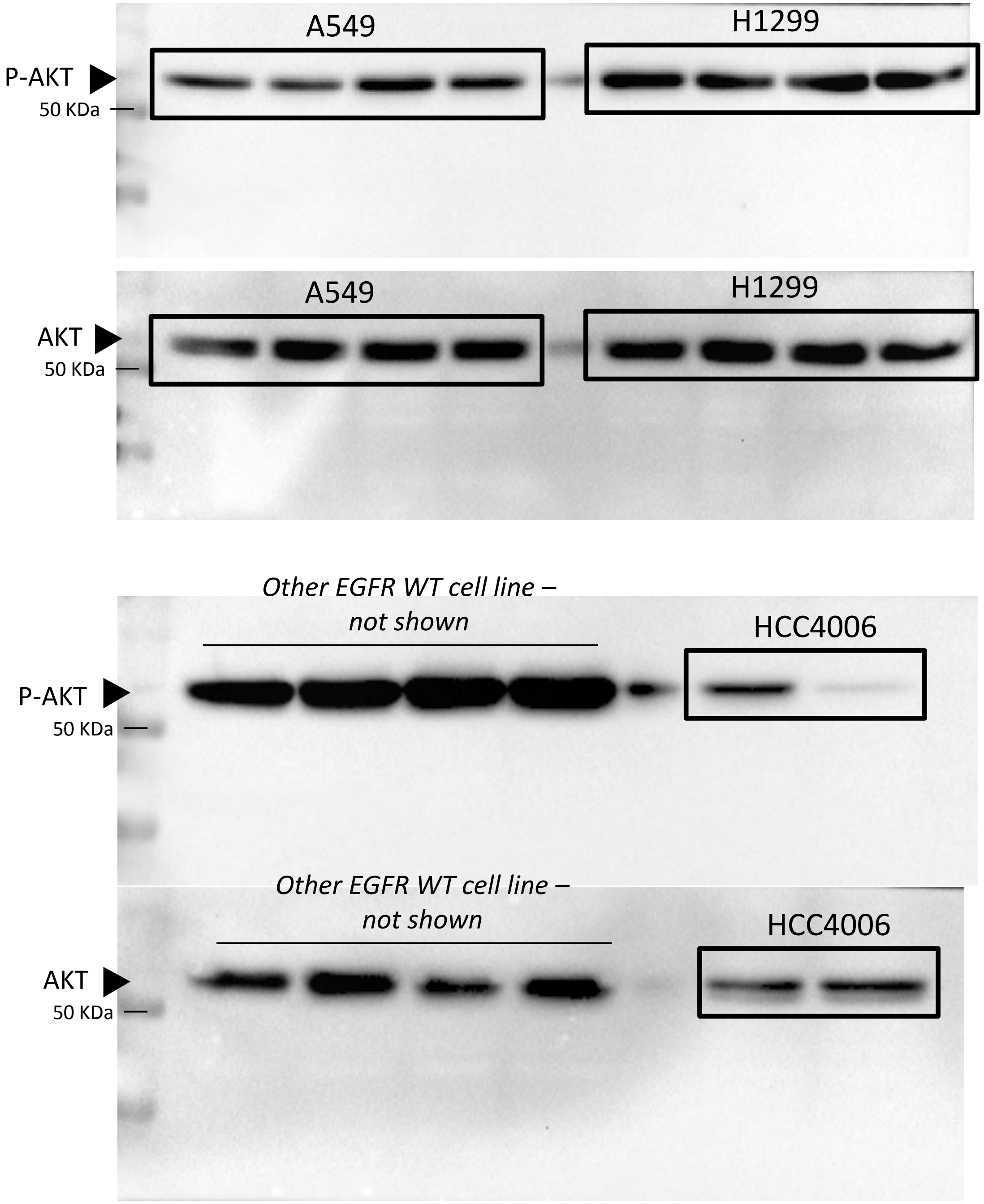
A549
H1299
P-AKT
50 KDa
A549
H1299
AKT
50 KDa
Other EGFR WT cell line –
not shown
HCC4006
P-AKT
50 KDa
Other EGFR WT cell line –
not shown
HCC4006
AKT
50 KDa

Fig. EV3B

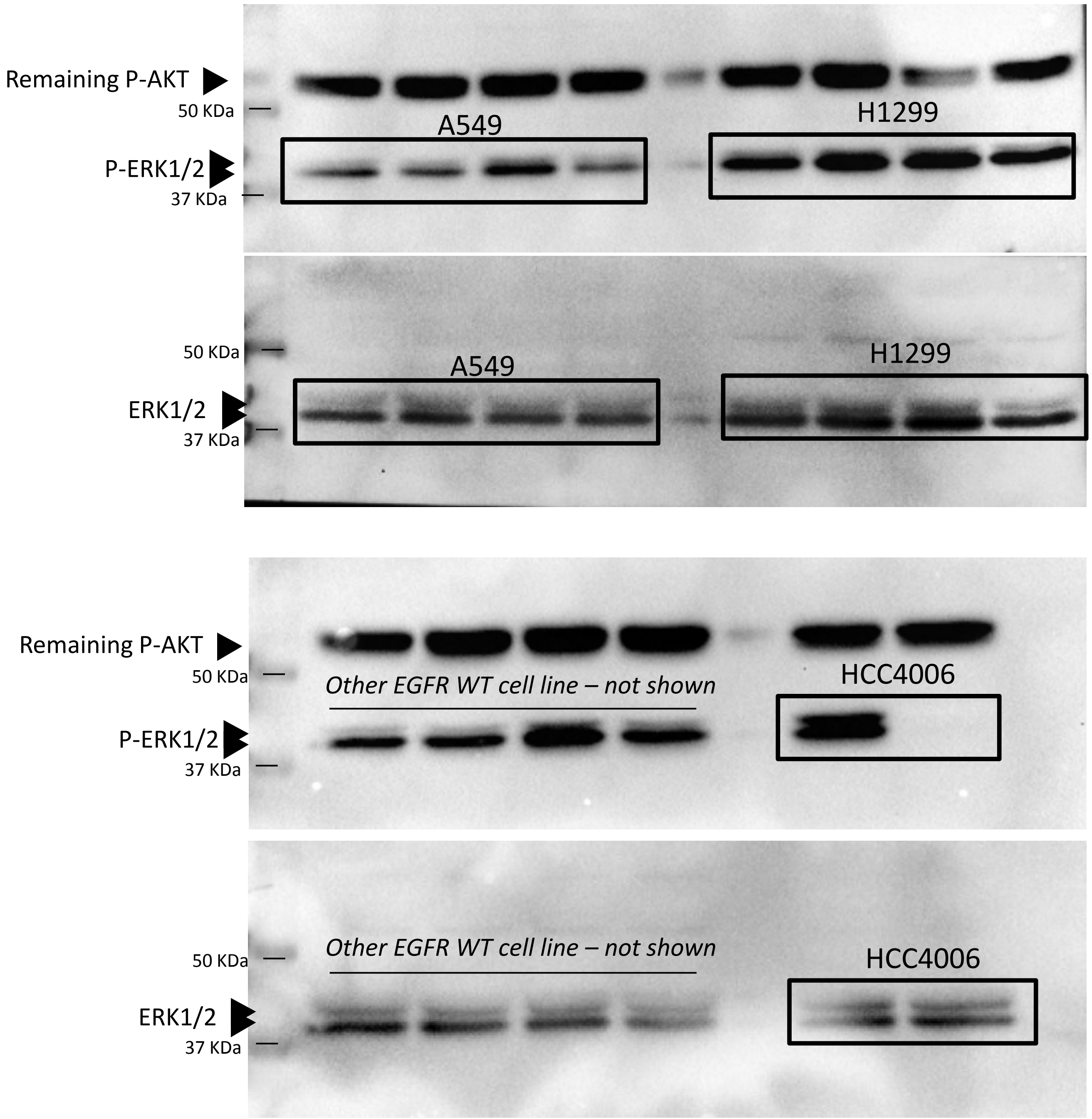
Remaining P-AKT
50 KDa
A549
H1299
P-ERK1/2
37 KDa
50 KDa
A549
H1299
ERK1/2
37 KDa
Remaining P-AKT
50 KDa
Other EGFR WT cell line – not shown
HCC4006
P-ERK1/2
37 KDa
50 KDa
Other EGFR WT cell line – not shown
HCC4006
ERK1/2
37 KDa

Fig. EV3B

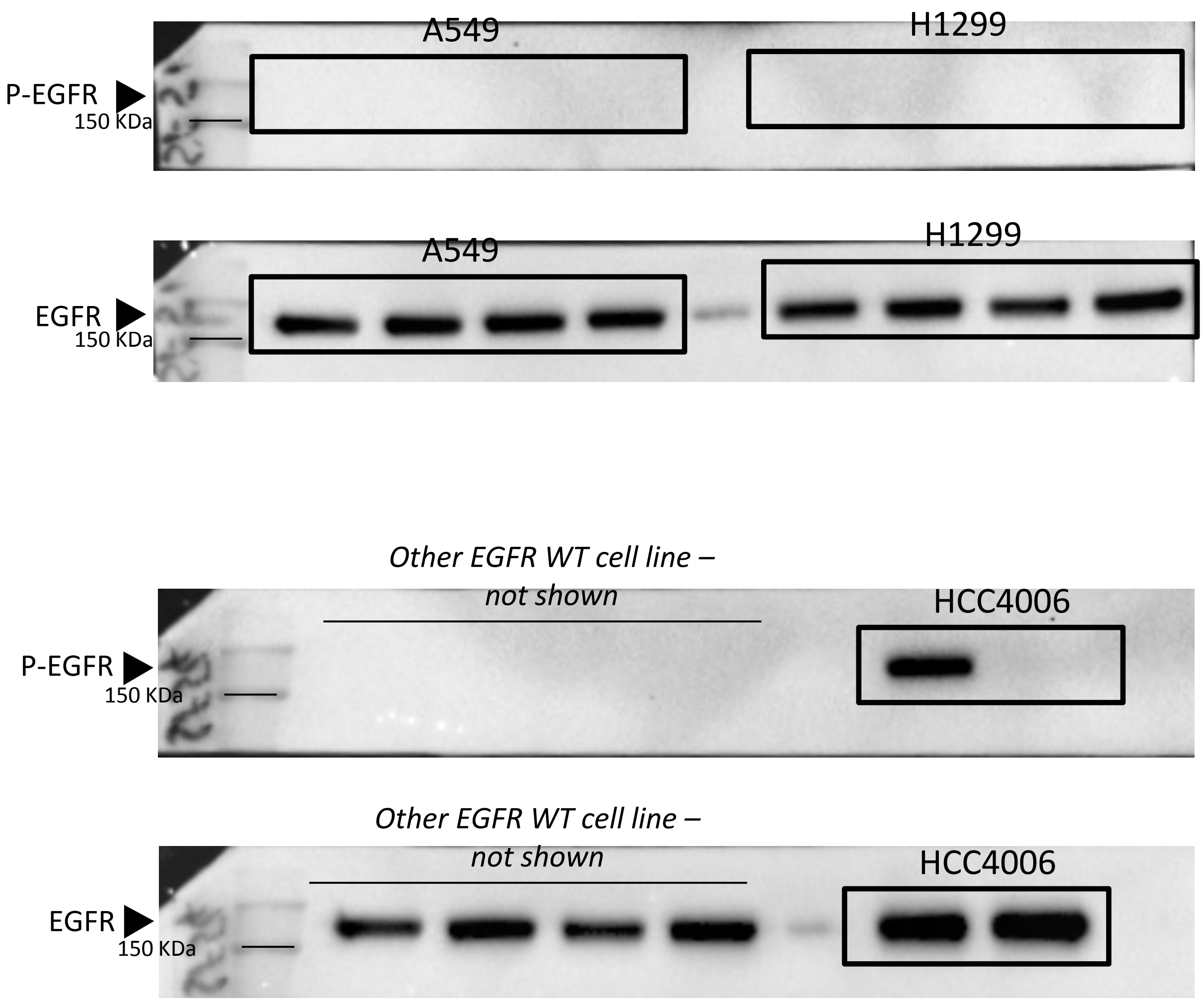
A549
H1299
P-EGFR
150 KDa
A549
H1299
EGFR
150 KDa
Other EGFR WT cell line –
not shown
HCC4006
P-EGFR
150 KDa
Other EGFR WT cell line –
not shown
HCC4006
EGFR
150 KDa

Fig. EV3B

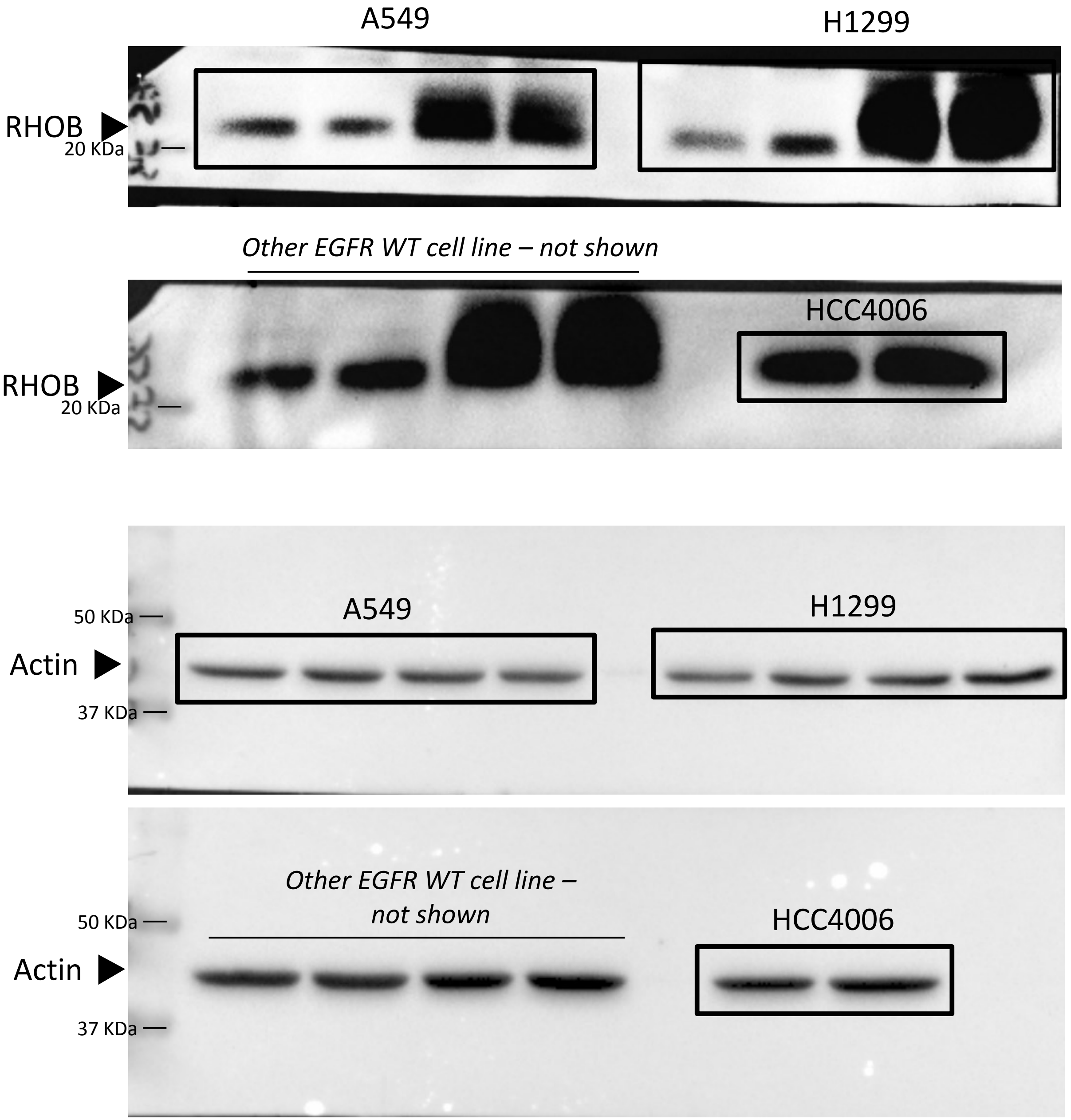
A549
H1299
RHOB
20 KDa
Other EGFR WT cell line – not shown
RHOB
20 KDa
HCC4006
A549
H1299
50 KDa
Actin
37 KDa
Other EGFR WT cell line – not shown
HCC4006
50 KDa
Actin
37 KDa
