# Supplementary figures and images for: The RAS‐related GTPase RHOB confers resistance to EGFR‐tyrosine kinase inhibitors in non‐small‐cell lung cancer via an AKT‐dependent mechanism
Source: EMBO Mol Med. 2016 Dec 22;9(2):238–50. doi: 10.15252/emmm.201606646 (PMC5286377; doi:10.15252/emmm.201606646)

Fig. EV2A

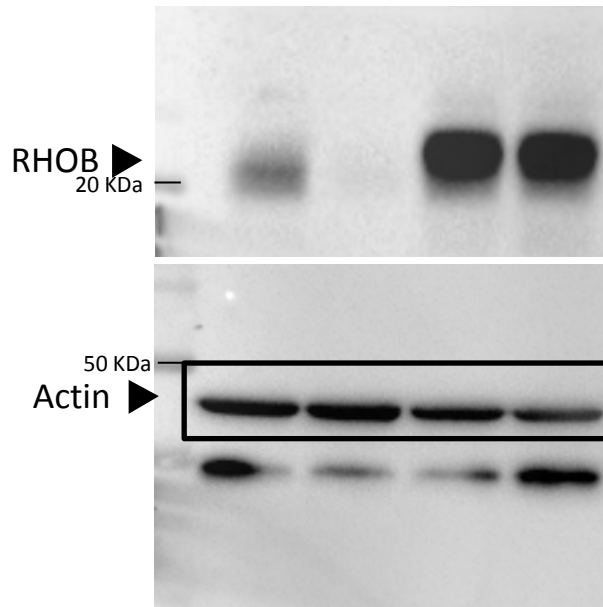

Fig. EV2B

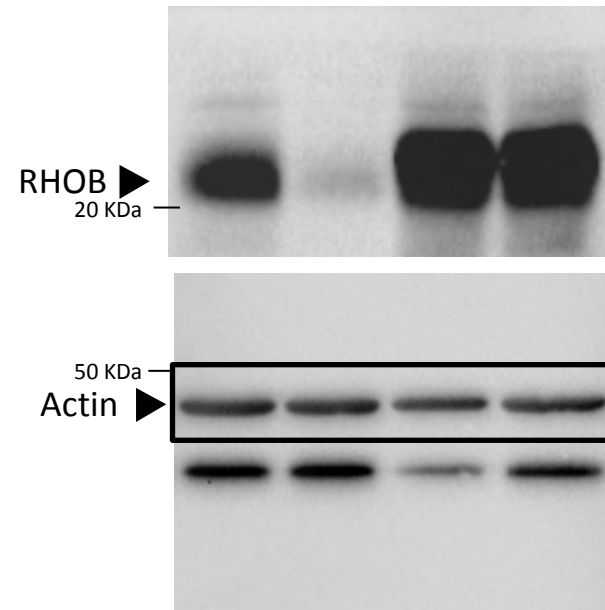

Fig. EV2C

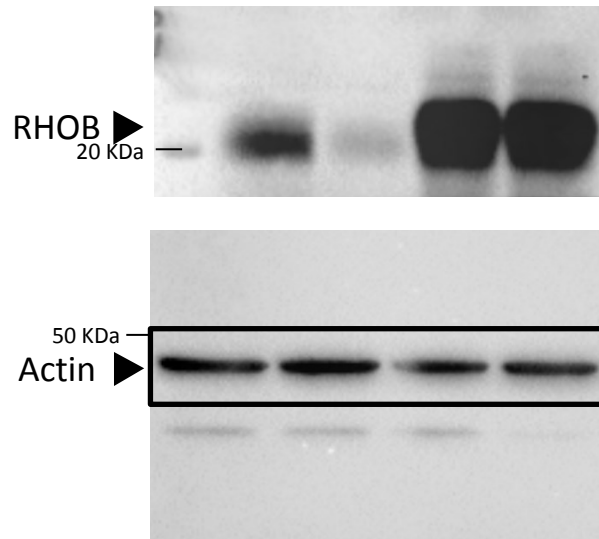

Fig. EV2D

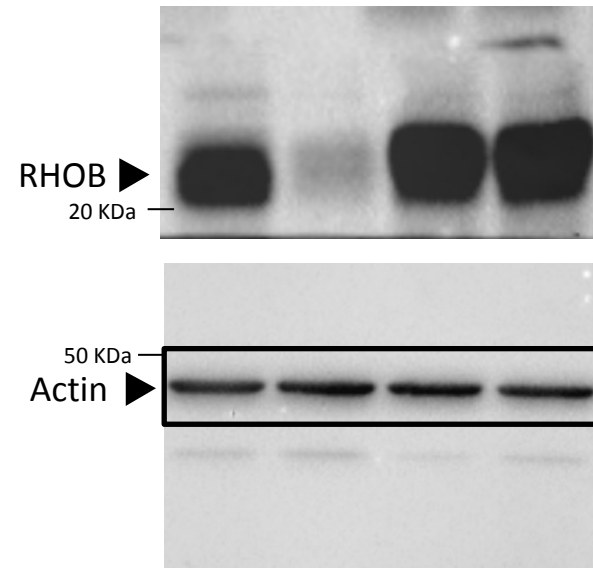

Supplement: Supplementary file 3 — Source Data for Expanded View and Appendix [file EMMM-9-238-s006.zip › EMM_06646_EV_Appendix_Source_Data/Figure_EV2/EMM_06646_Fig_EV2_source_data.pdf]

Fig. EV4A

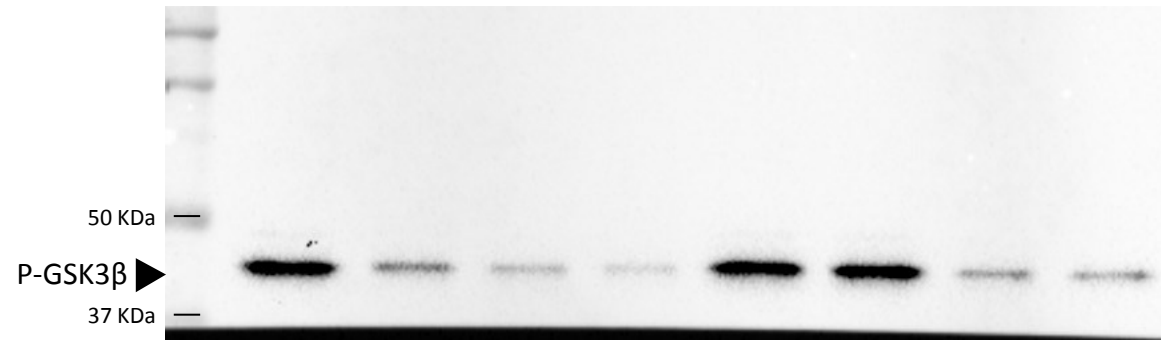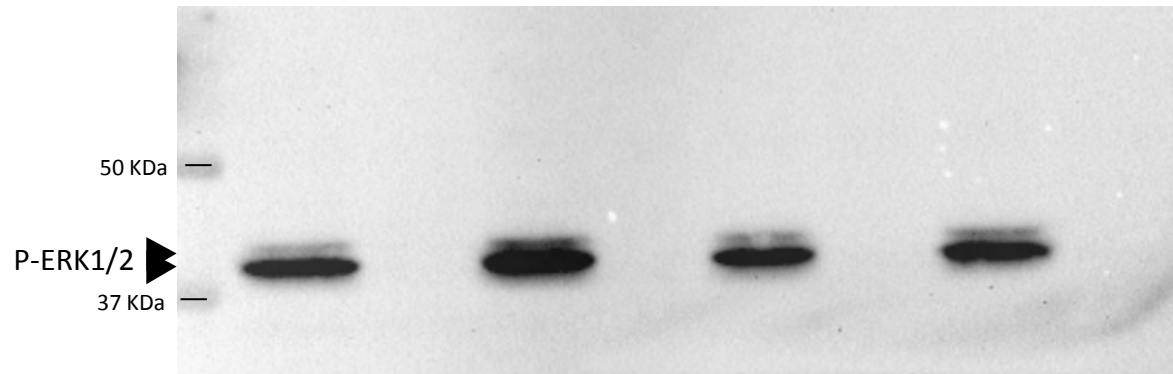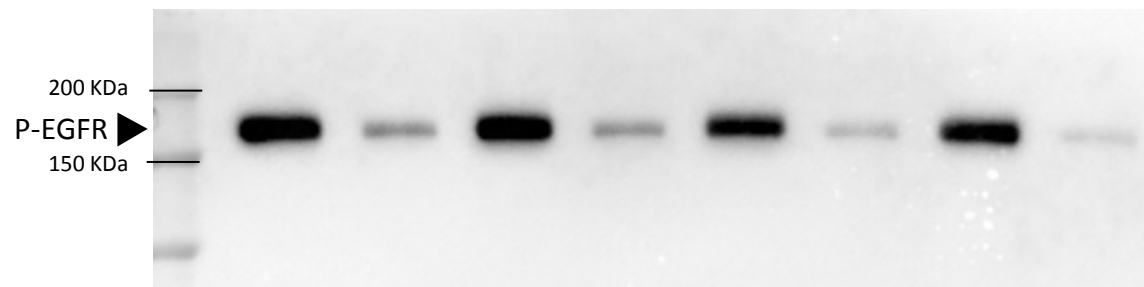

Fig. EV4A

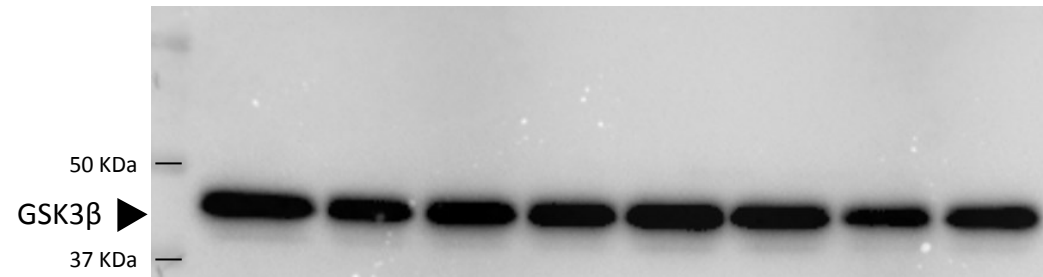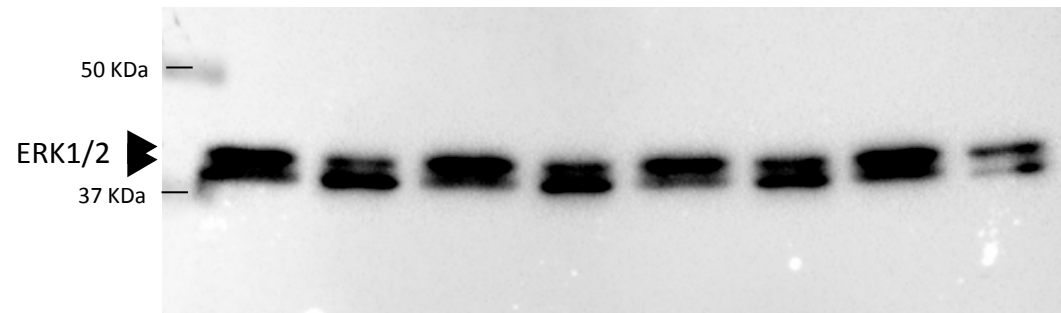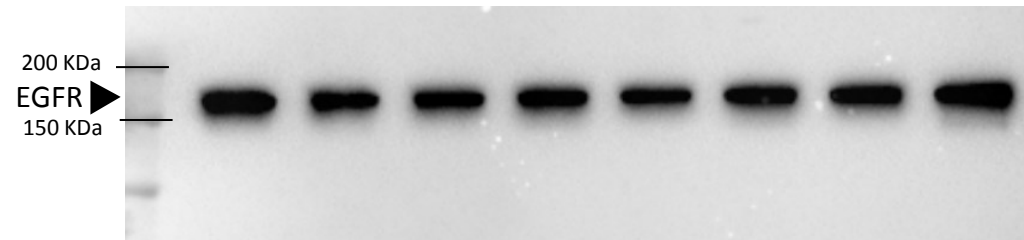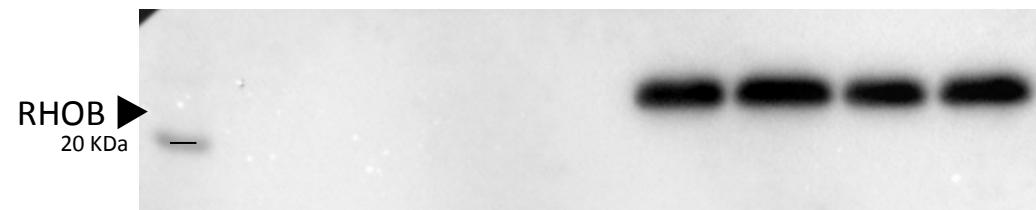

Fig. EV4B

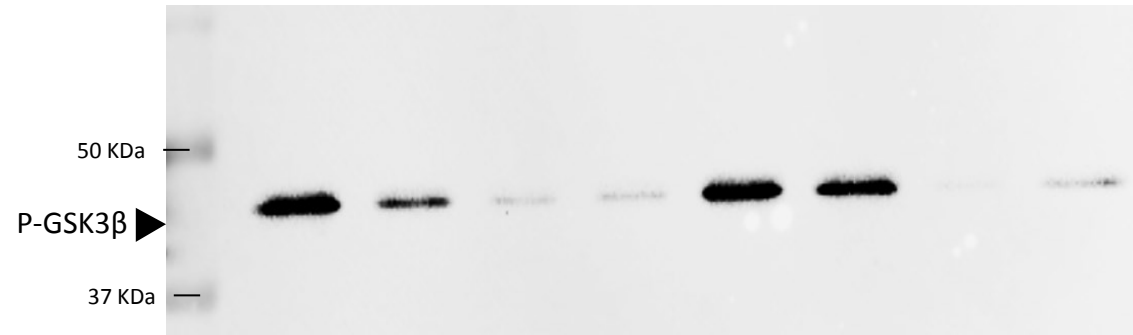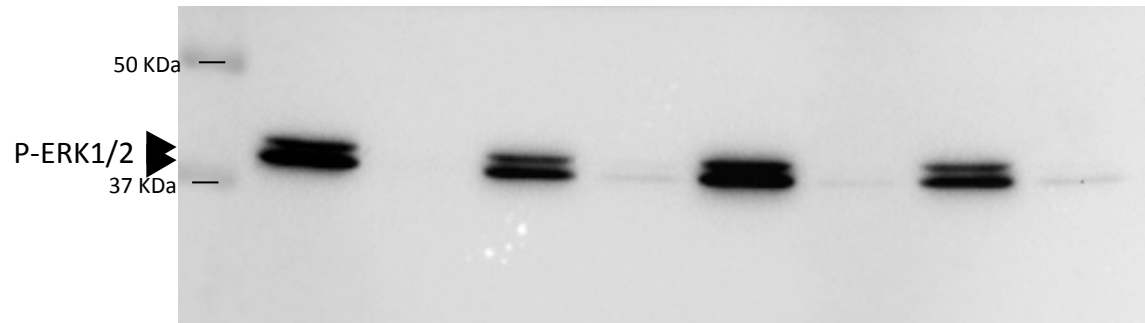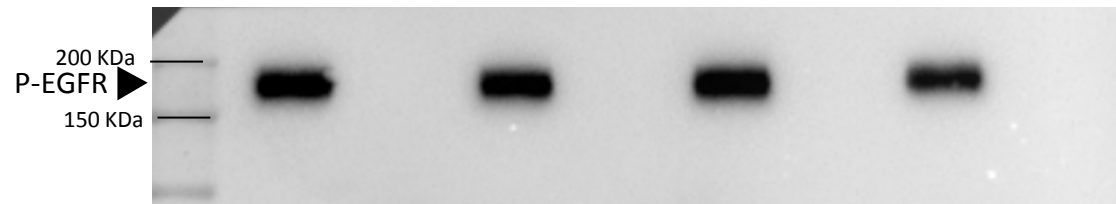

Fig. EV4B

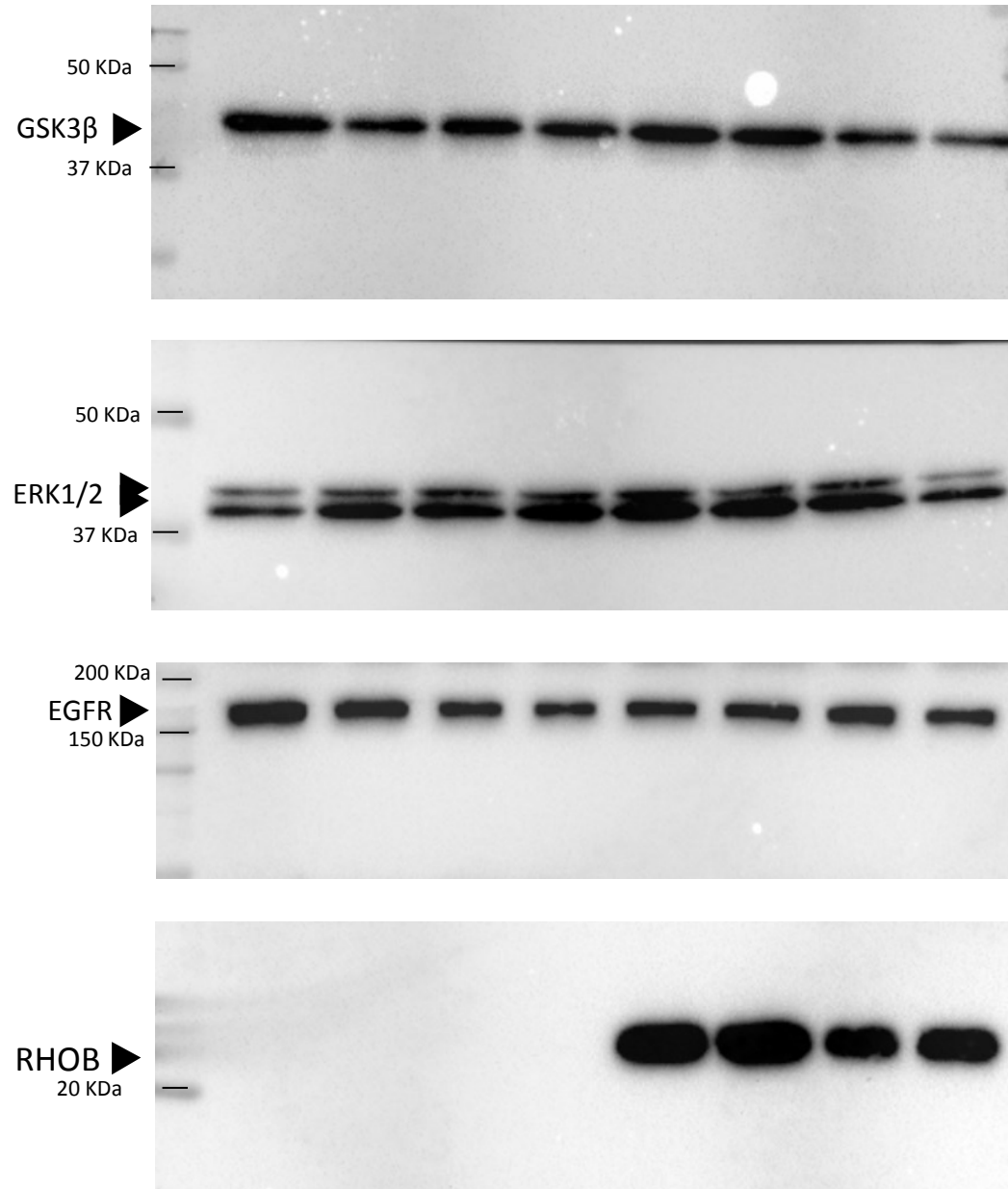

Fig. EV4C

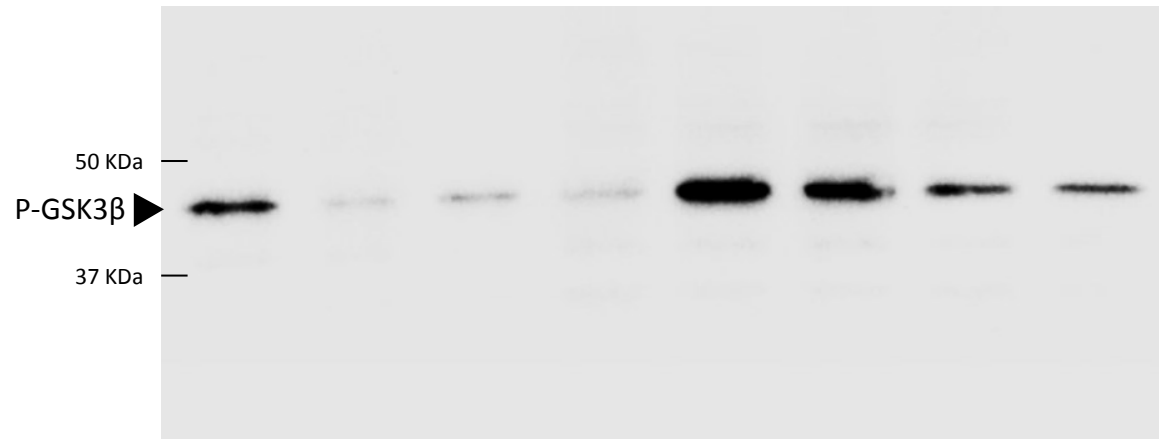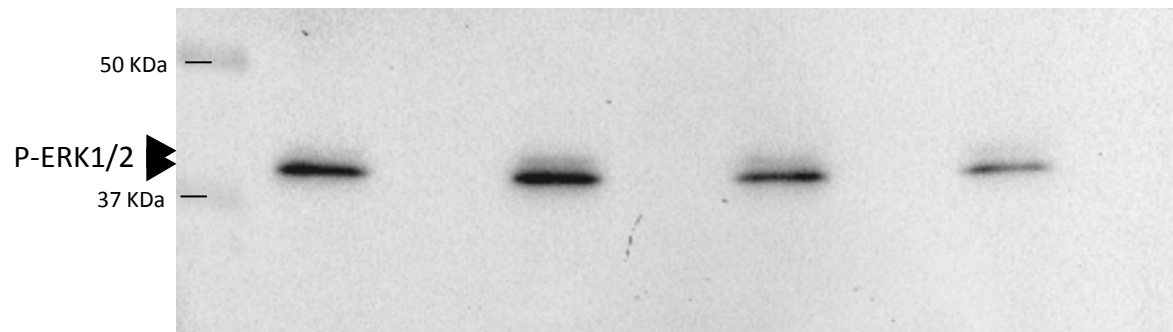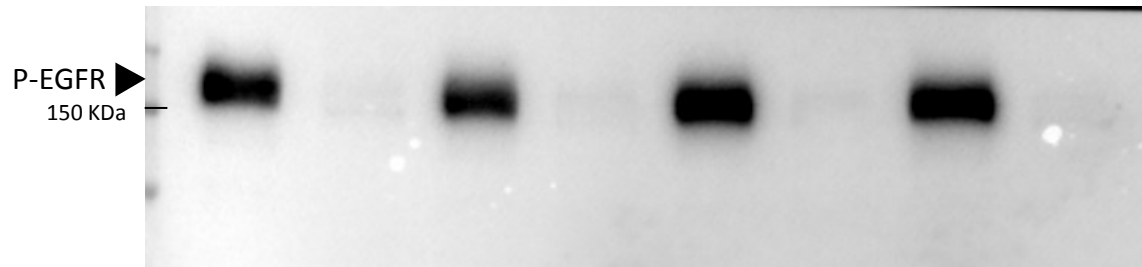

Fig. EV4C

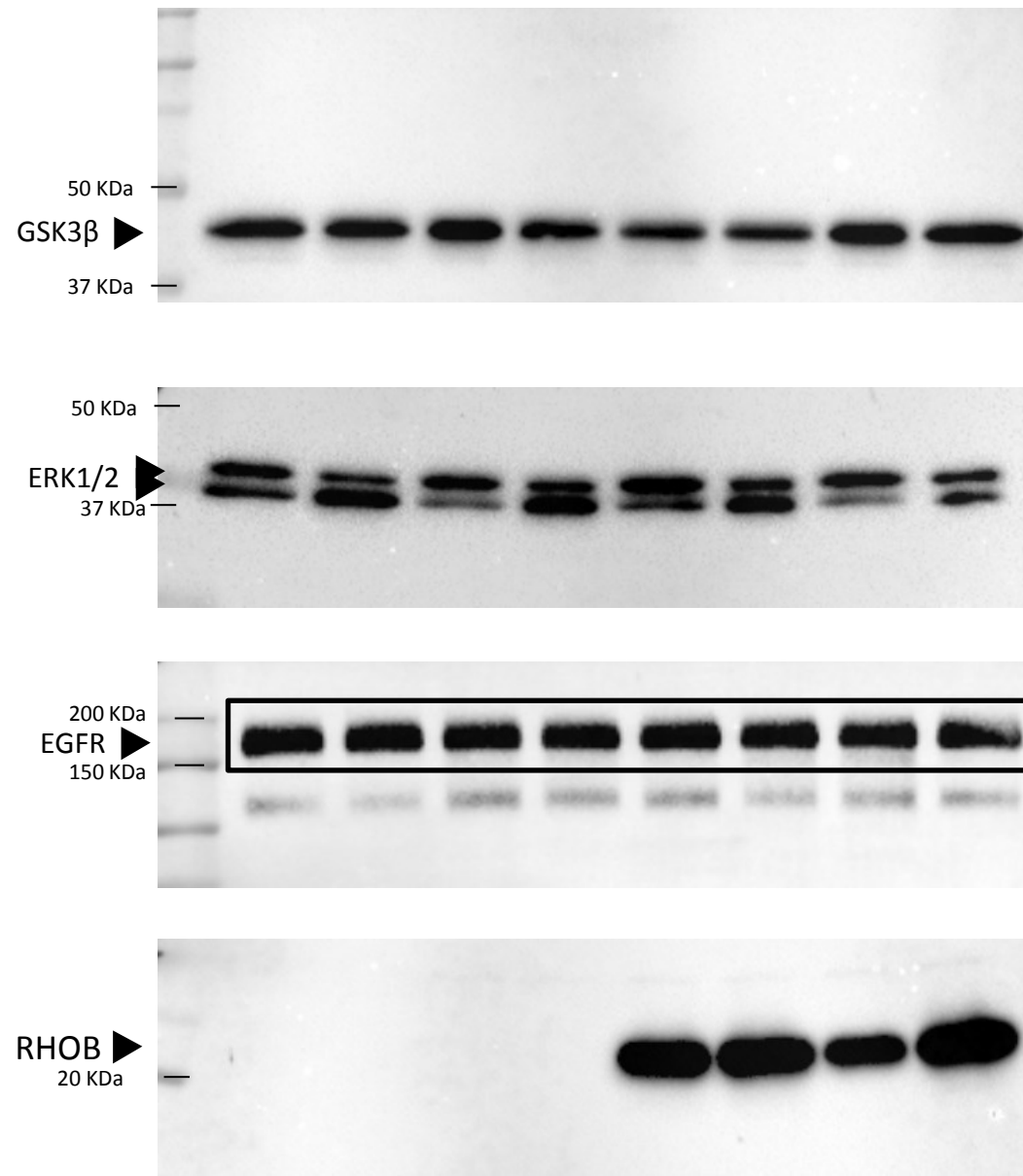

Supplement: Supplementary file 3 — Source Data for Expanded View and Appendix [file EMMM-9-238-s006.zip › EMM_06646_EV_Appendix_Source_Data/Figure_EV4/EMM_06646_Fig_EV4_source_data.pdf]

Fig. EV5A

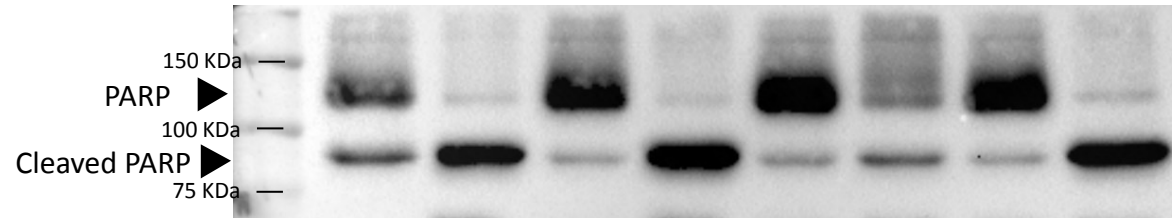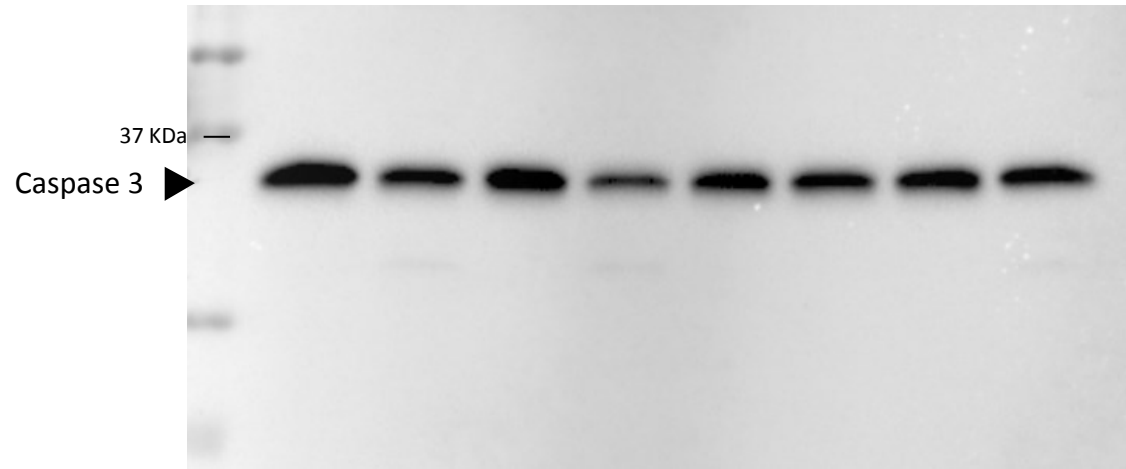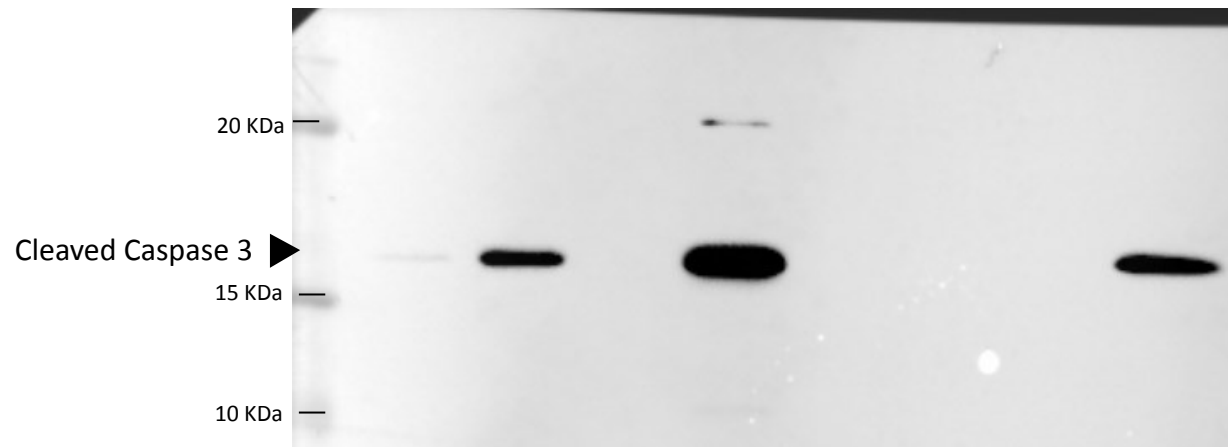

Fig. EV5B

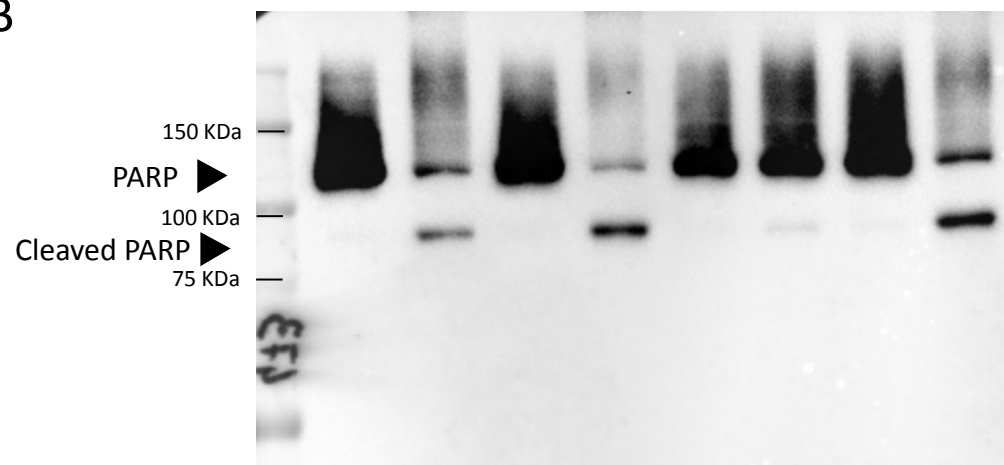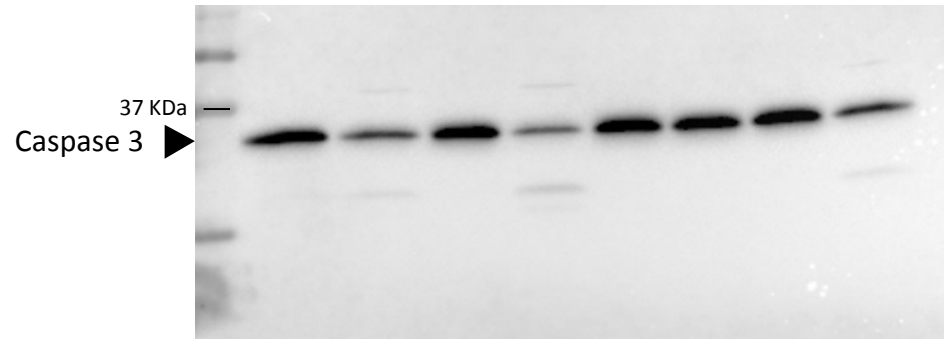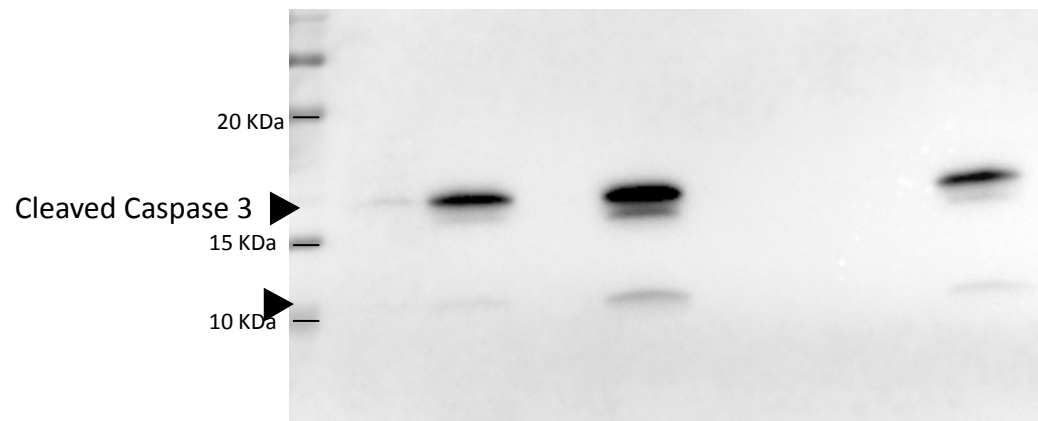

Fig. EV5C

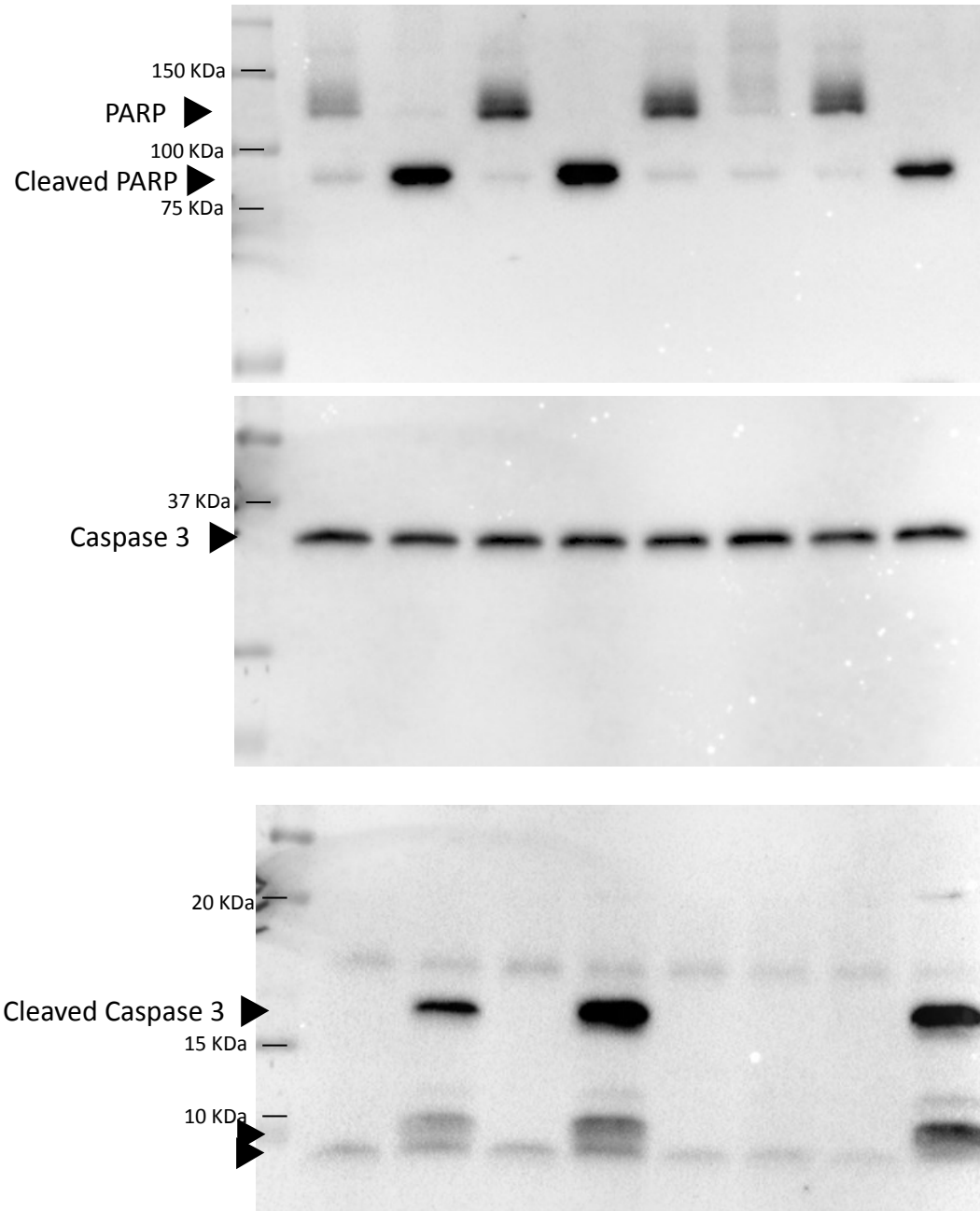

Supplement: Supplementary file 3 — Source Data for Expanded View and Appendix [file EMMM-9-238-s006.zip › EMM_06646_EV_Appendix_Source_Data/Figure_EV5/EMM_06646_Fig_EV5_source_data.pdf]

Appendix Figure S1C

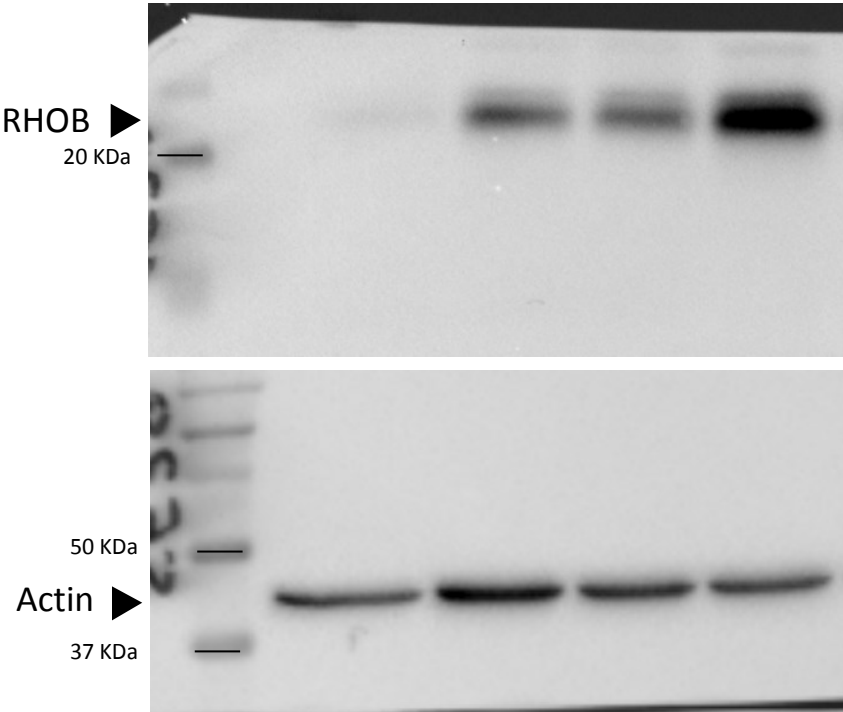

Supplement: Supplementary file 3 — Source Data for Expanded View and Appendix [file EMMM-9-238-s006.zip › EMM_06646_EV_Appendix_Source_Data/Figure_S1/EMM_06646_Fig_S1_source_data.pdf]

Appendix Figure S2

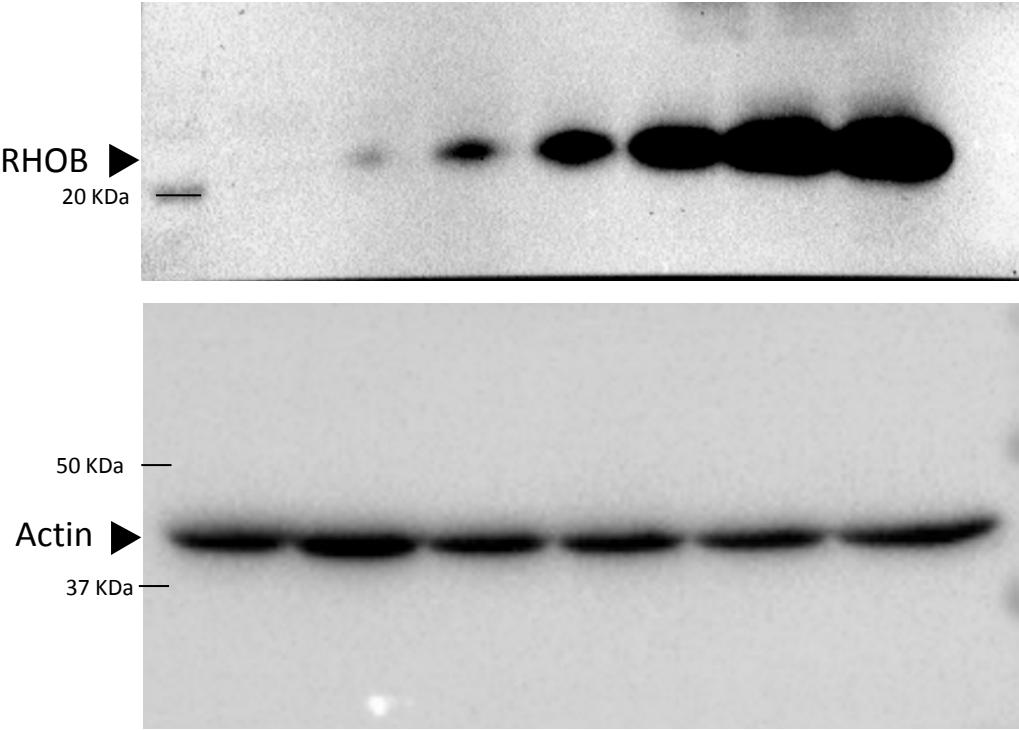

Supplement: Supplementary file 3 — Source Data for Expanded View and Appendix [file EMMM-9-238-s006.zip › EMM_06646_EV_Appendix_Source_Data/Figure_S2/EMM_06646_Fig_S2_source_data.pdf]

Appendix Figure S3

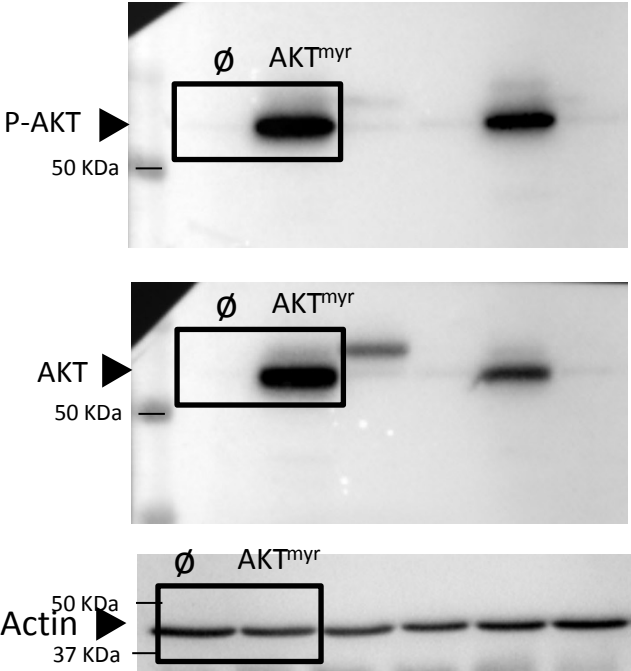

Supplement: Supplementary file 3 — Source Data for Expanded View and Appendix [file EMMM-9-238-s006.zip › EMM_06646_EV_Appendix_Source_Data/Figure_S3/EMM_06646_Fig_S3_source_data.pdf]

Appendix Figure S7

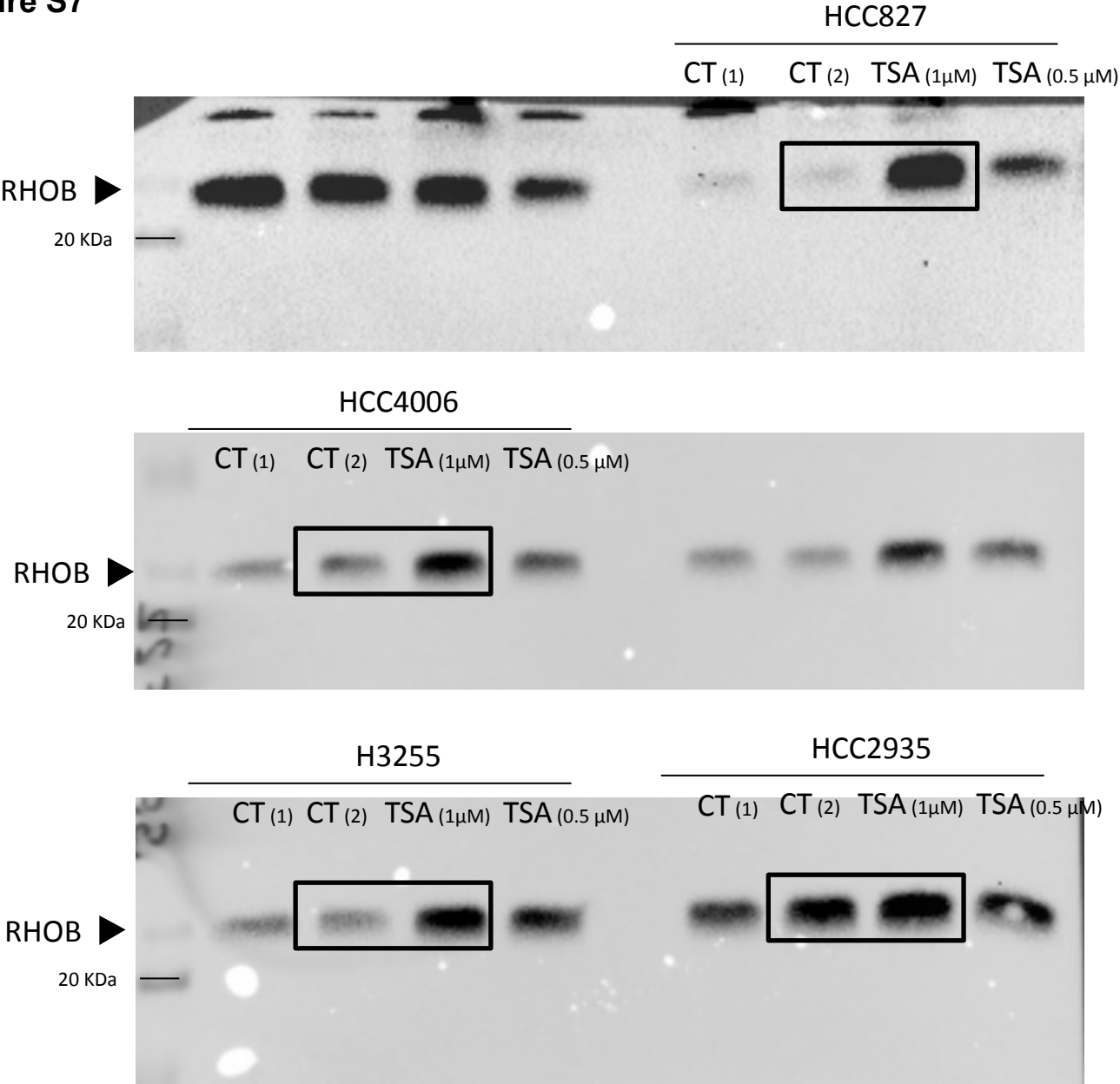

Appendix Figure S7

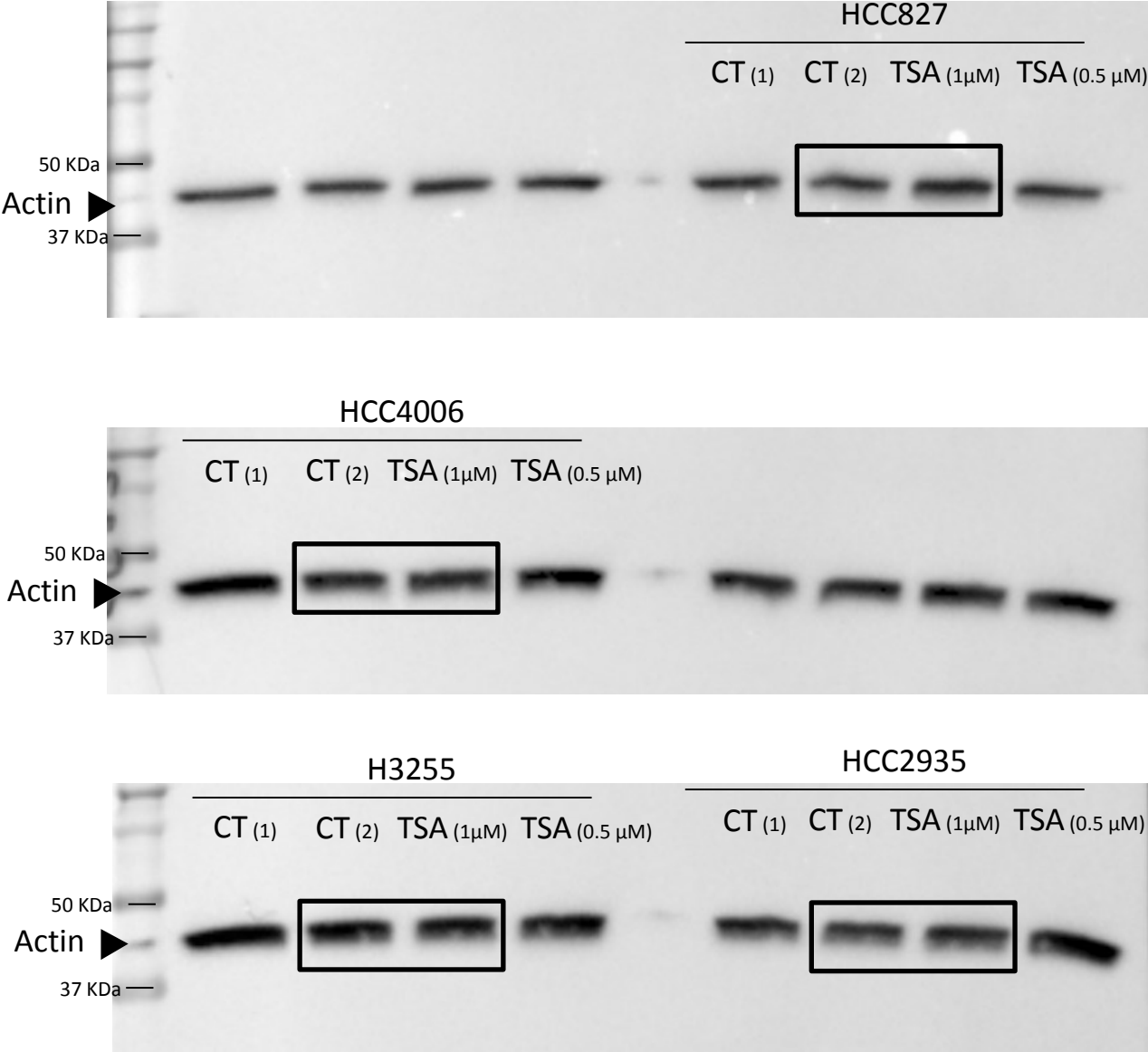

Supplement: Supplementary file 3 — Source Data for Expanded View and Appendix [file EMMM-9-238-s006.zip › EMM_06646_EV_Appendix_Source_Data/Figure_S7/EMM_06646_Fig_S7_source_data.pdf]

Fig. 3A

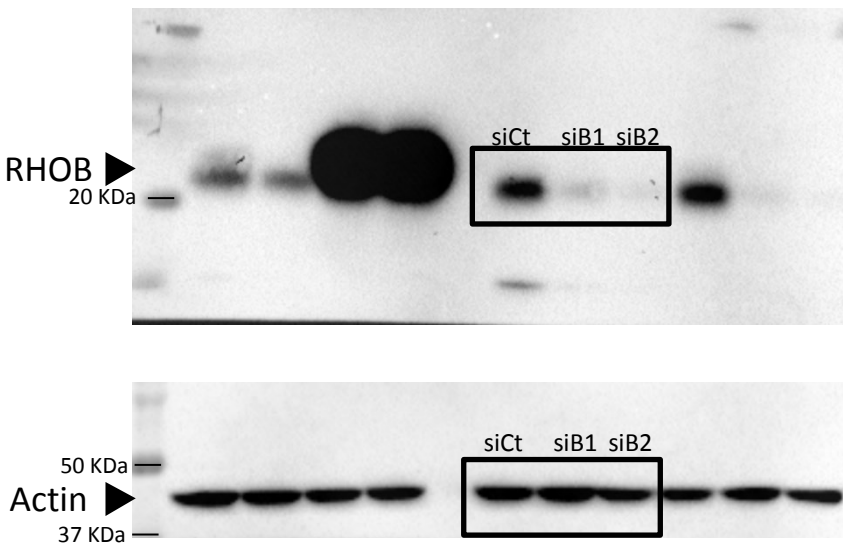

Fig. 3B

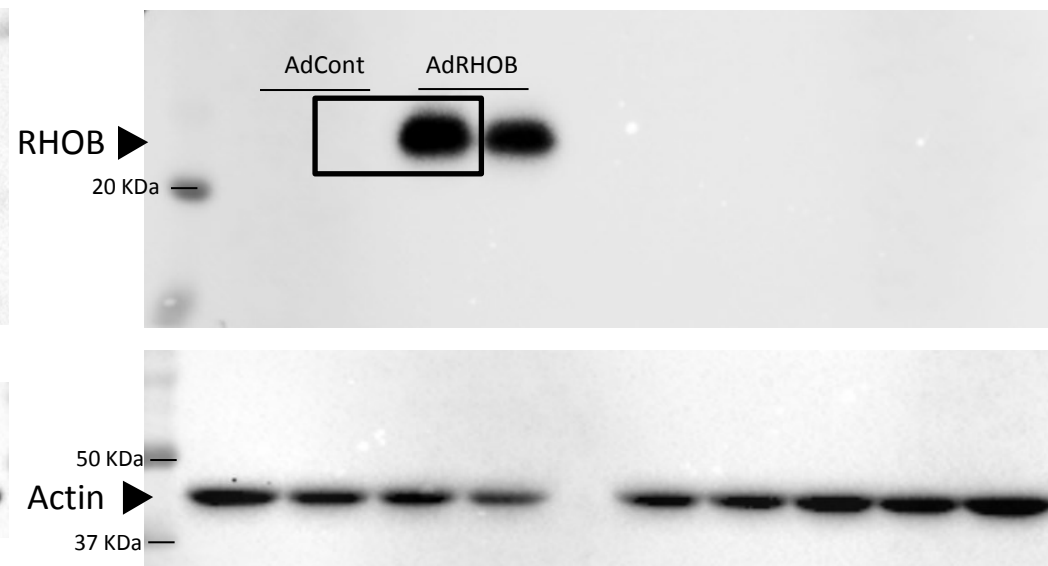

Fig. 3C

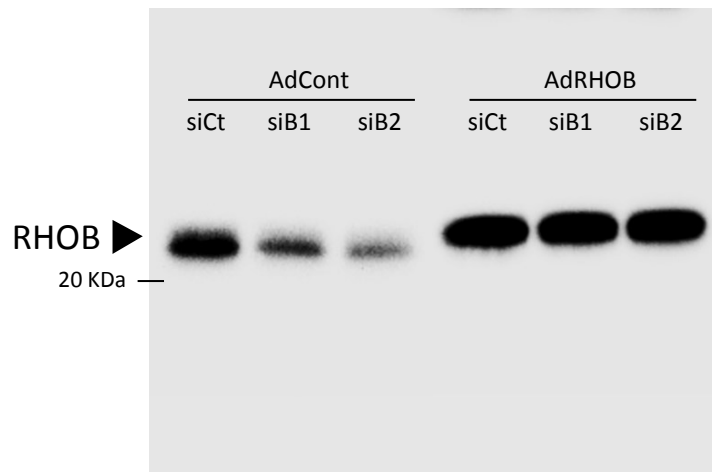

Fig. 3C

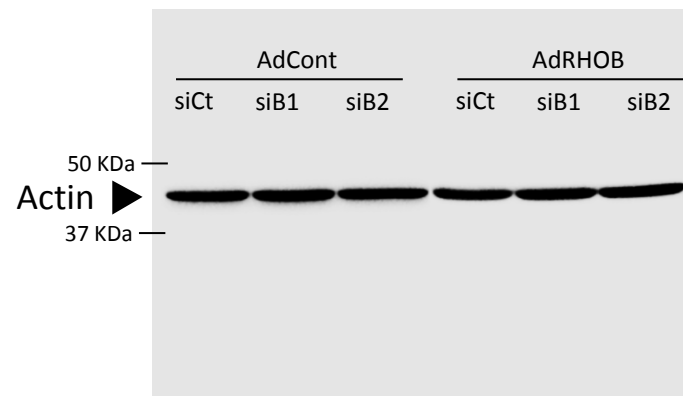

Fig. 3Da

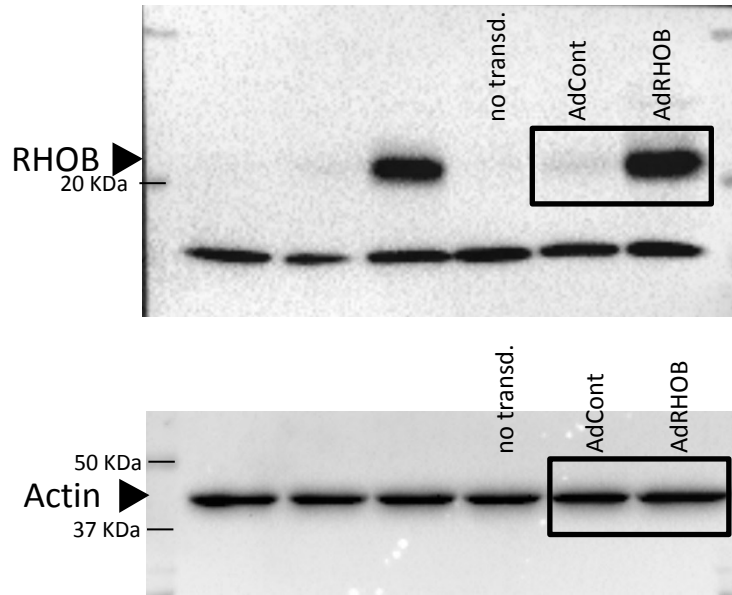

Fig. 3Db

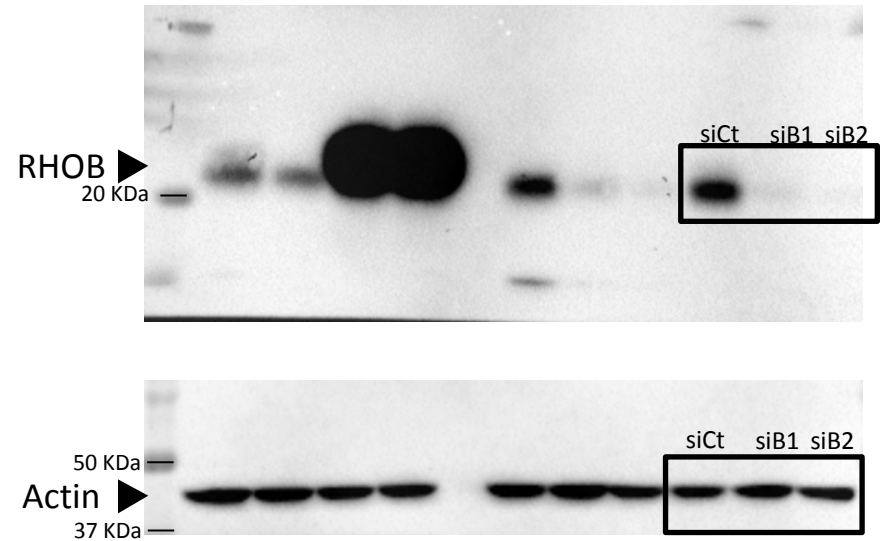

Fig. 3Ea

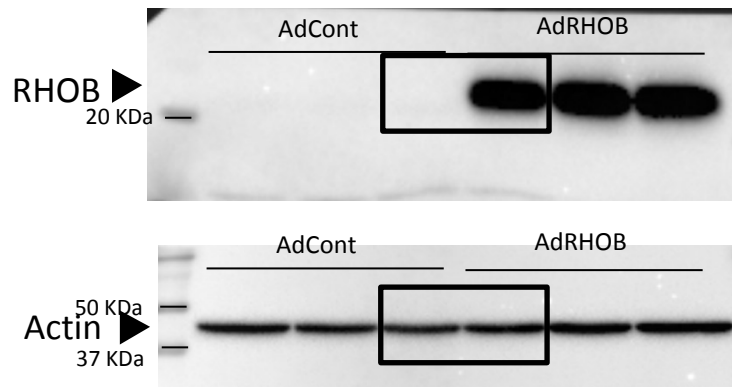

Fig. 3Eb

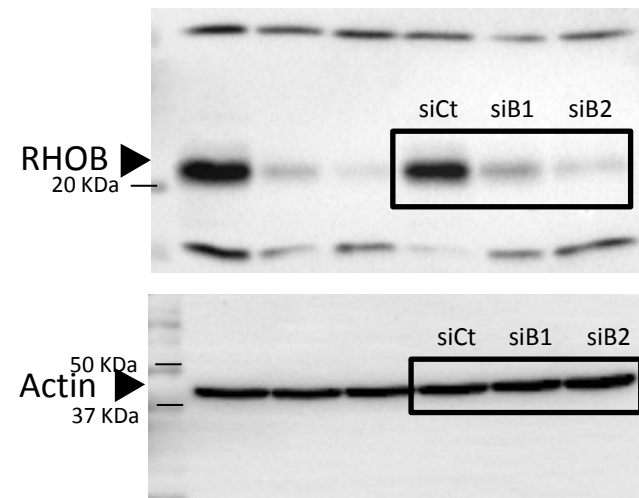

Fig. 3Fa

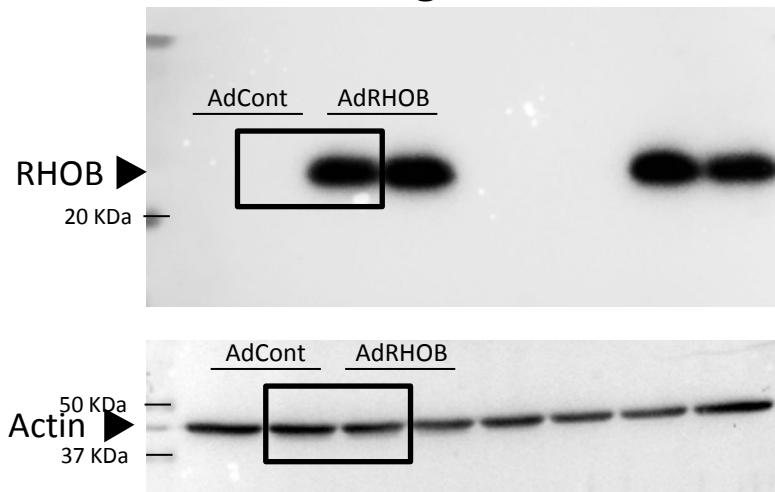

Fig. 3Fb

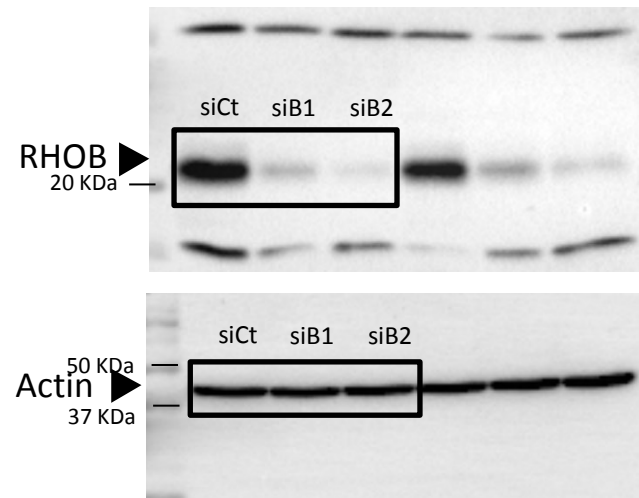

Fig. 3G

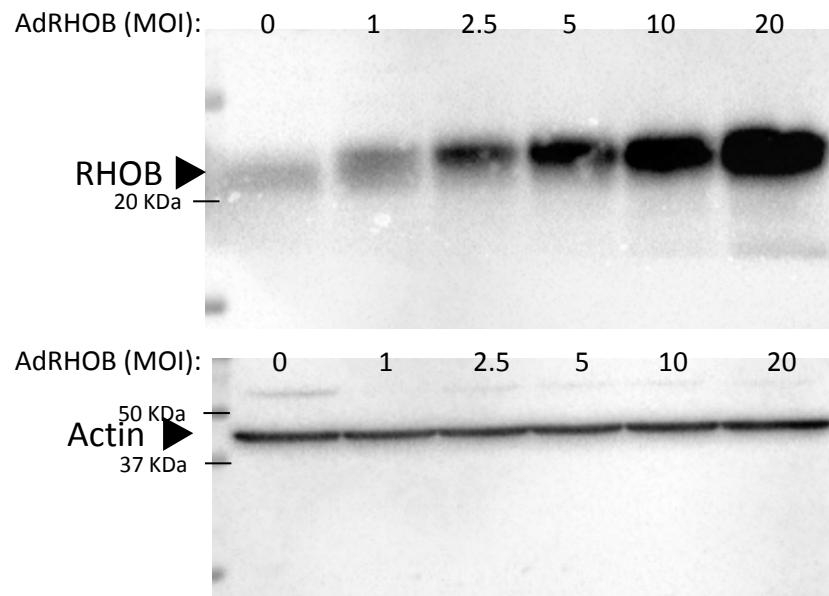

Supplement: Supplementary file 5 — Source Data for Figure 3 [file EMMM-9-238-s003.pdf]

Fig. 5A

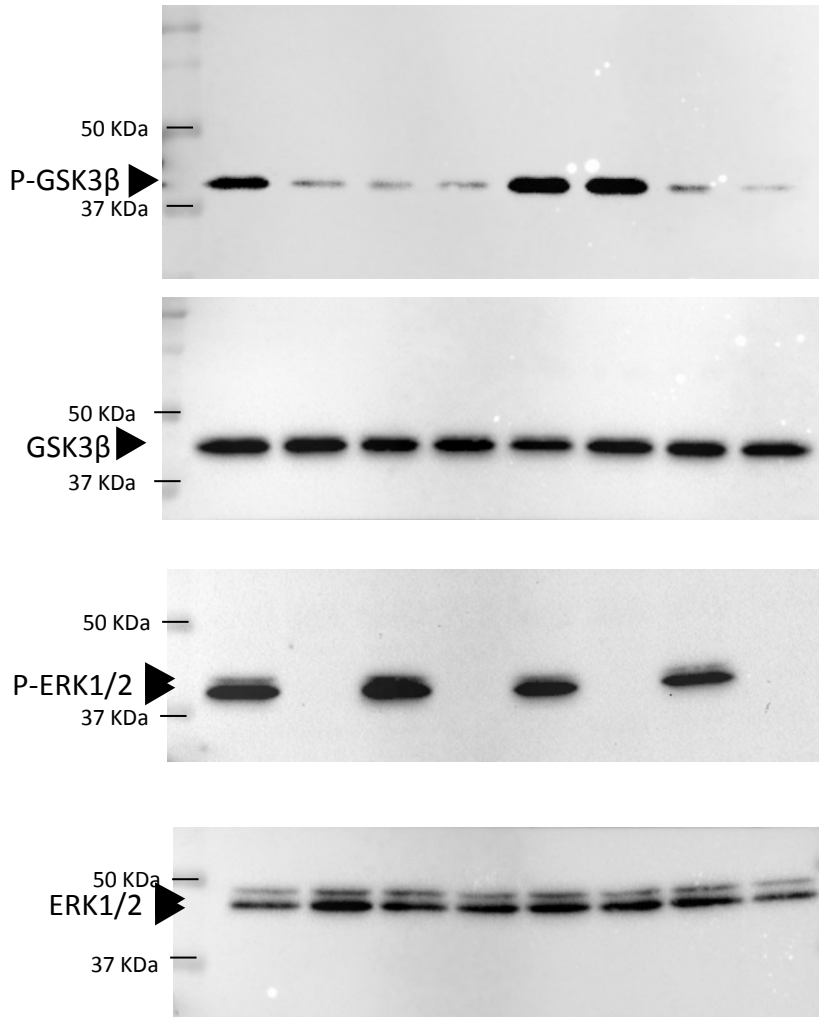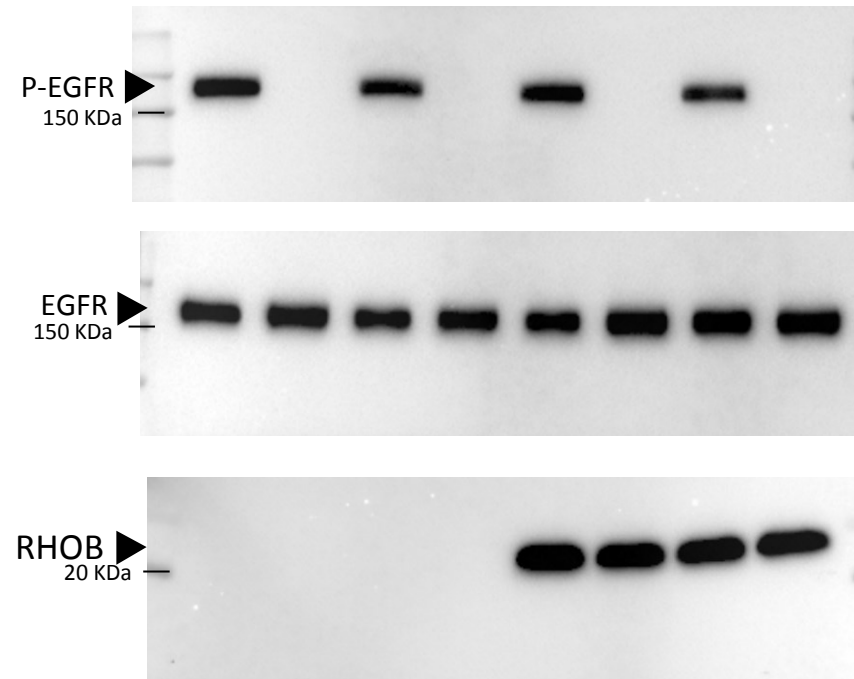

Fig. 5E

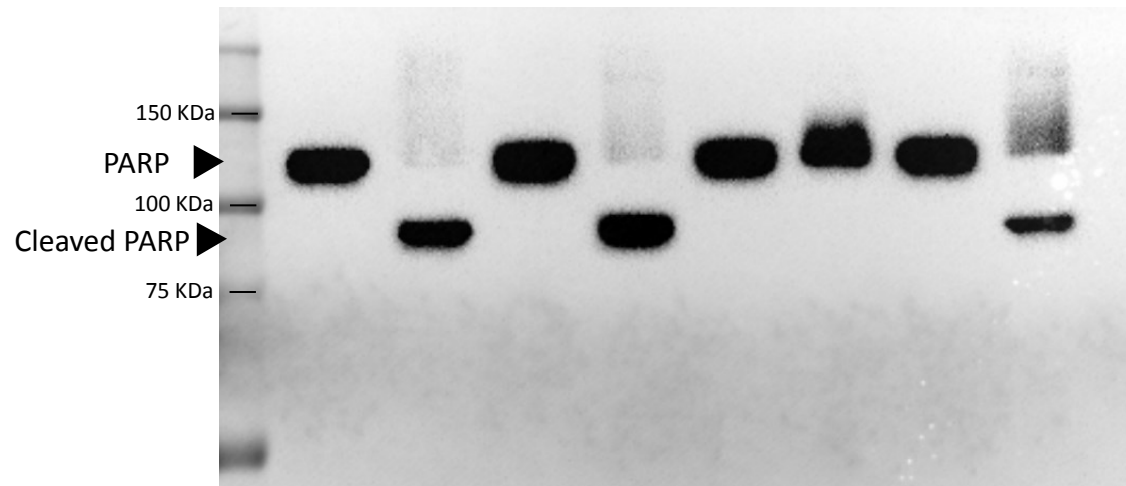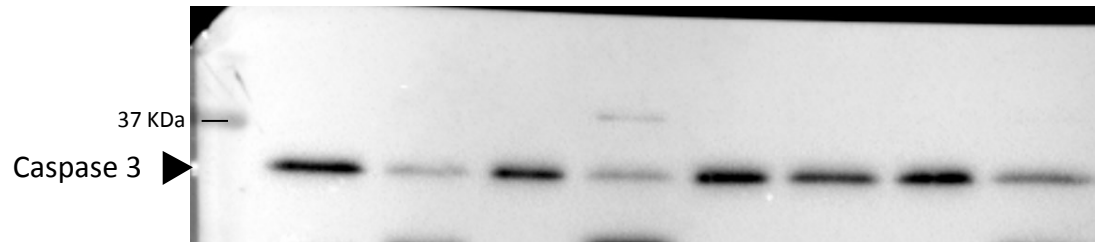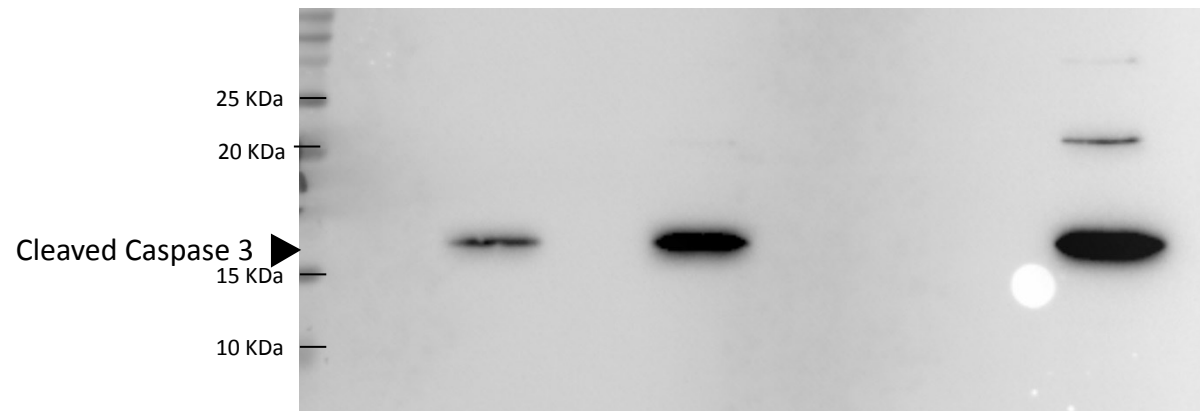

Supplement: Supplementary file 7 — Source Data for Figure 5 [file EMMM-9-238-s005.pdf]
