# Supplementary material for: The RAS‐related GTPase RHOB confers resistance to EGFR‐tyrosine kinase inhibitors in non‐small‐cell lung cancer via an AKT‐dependent mechanism
Source: EMBO Mol Med. 2016 Dec 22;9(2):238–50. doi: 10.15252/emmm.201606646 (PMC5286377; doi:10.15252/emmm.201606646)
Supplement: Supplementary file 6 — Source Data for Figure 4 [file EMMM-9-238-s004.pdf]

Fig. 4Aa

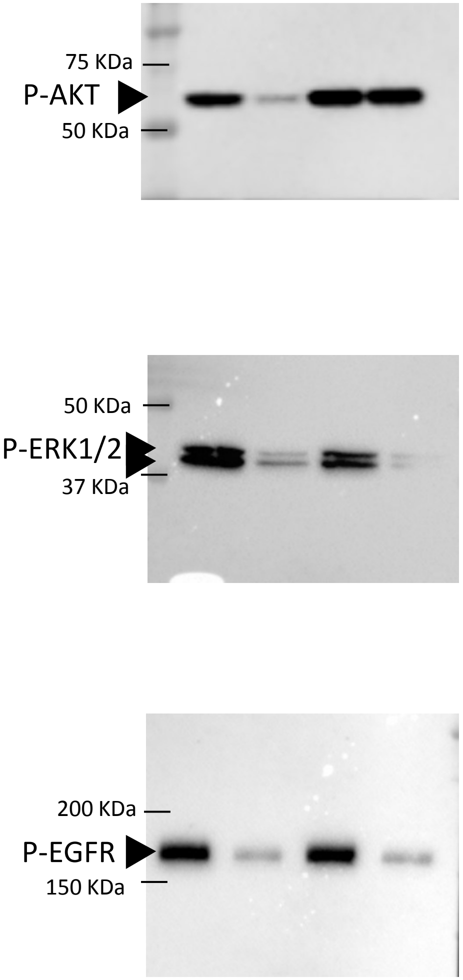

Fig. 4Ab

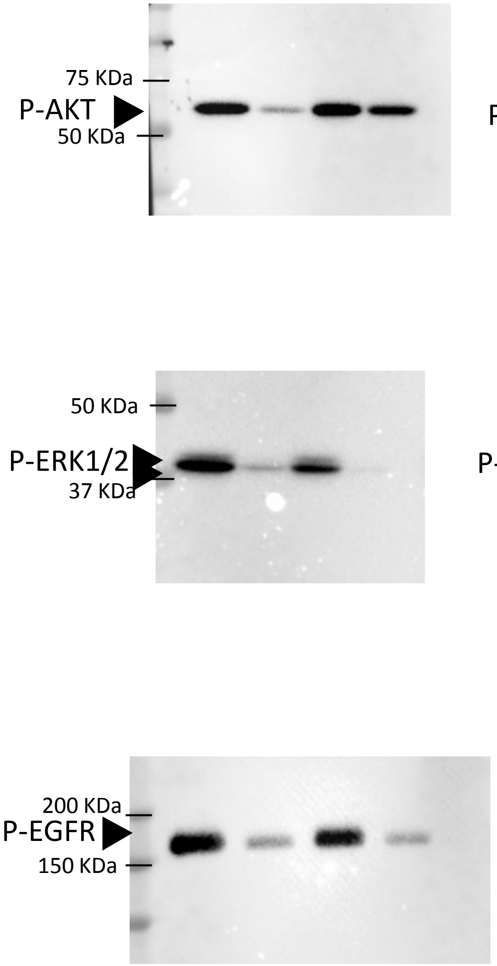

Fig. 4Ac

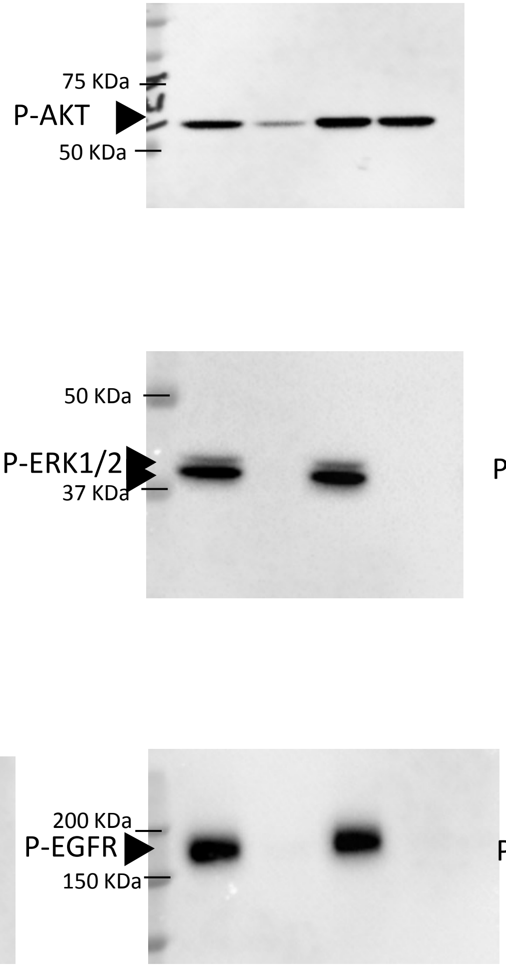

Fig. 4Ad

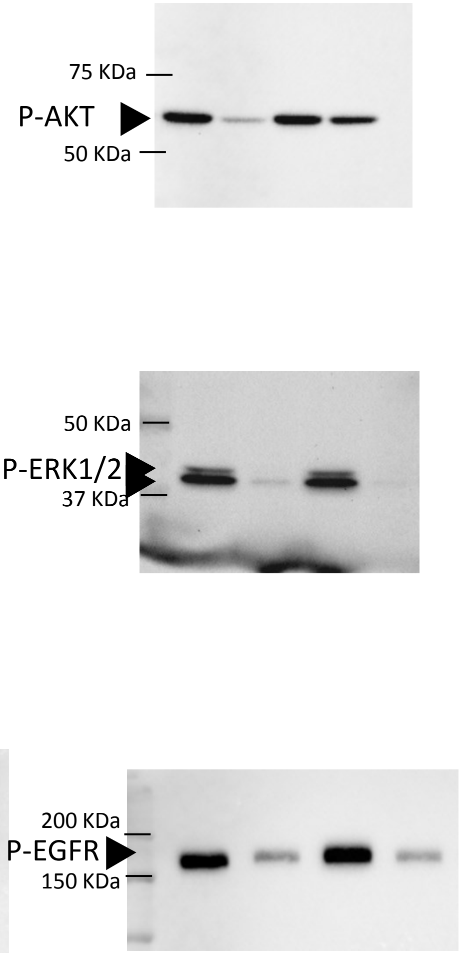

Fig. 4Aa

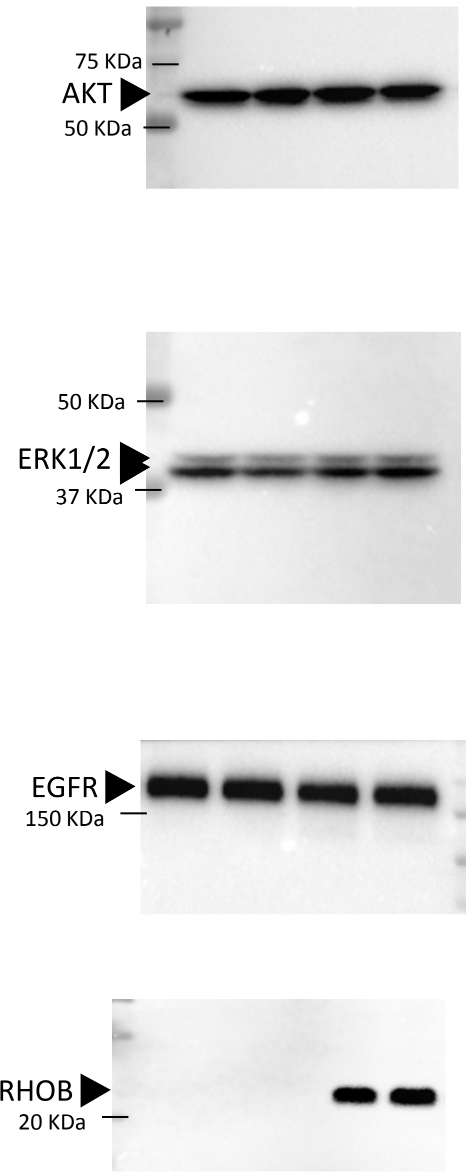

Fig. 4Ab

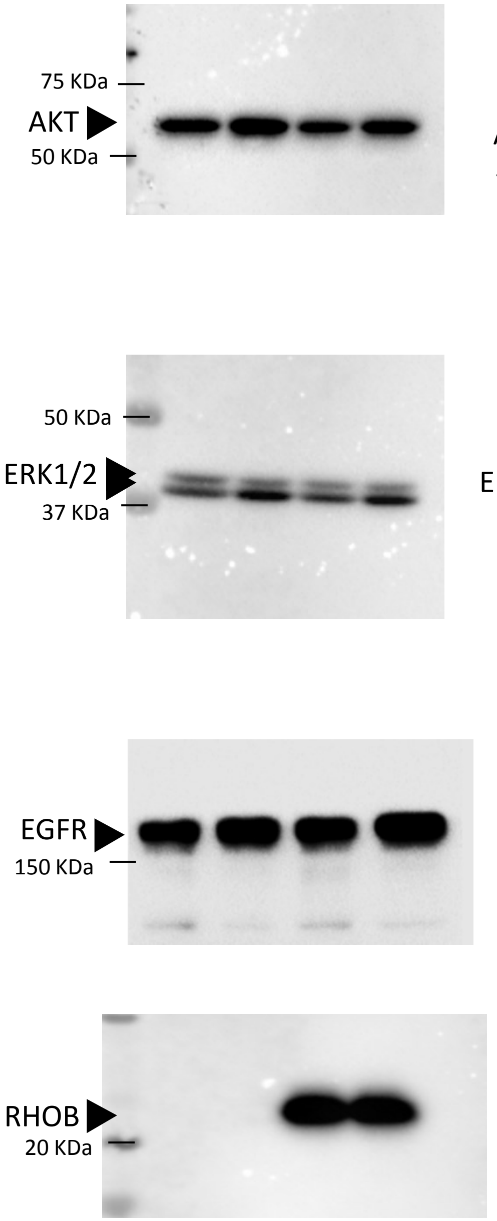

Fig. 4Ac

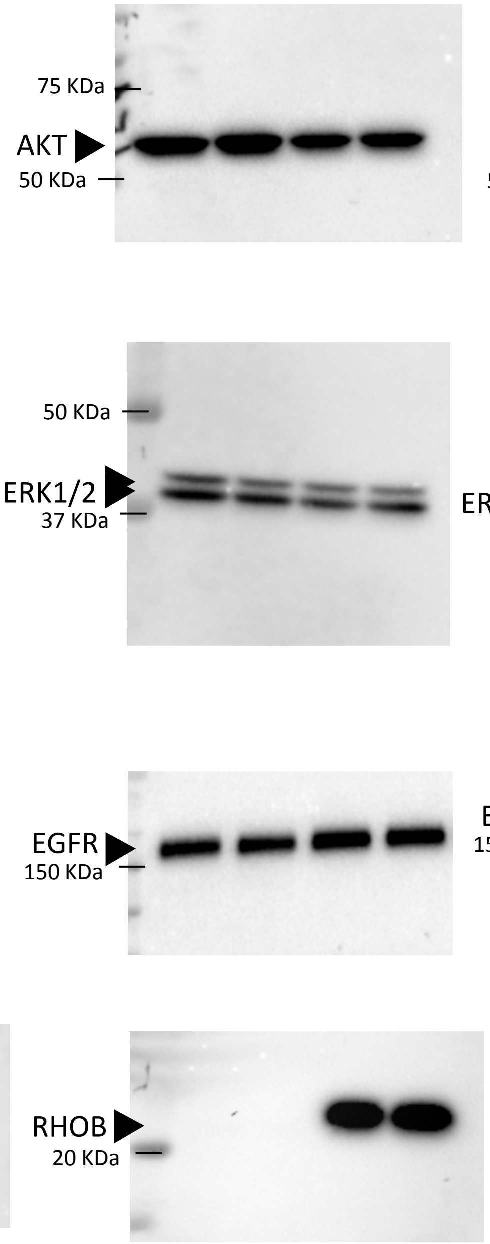

Fig. 4Ad

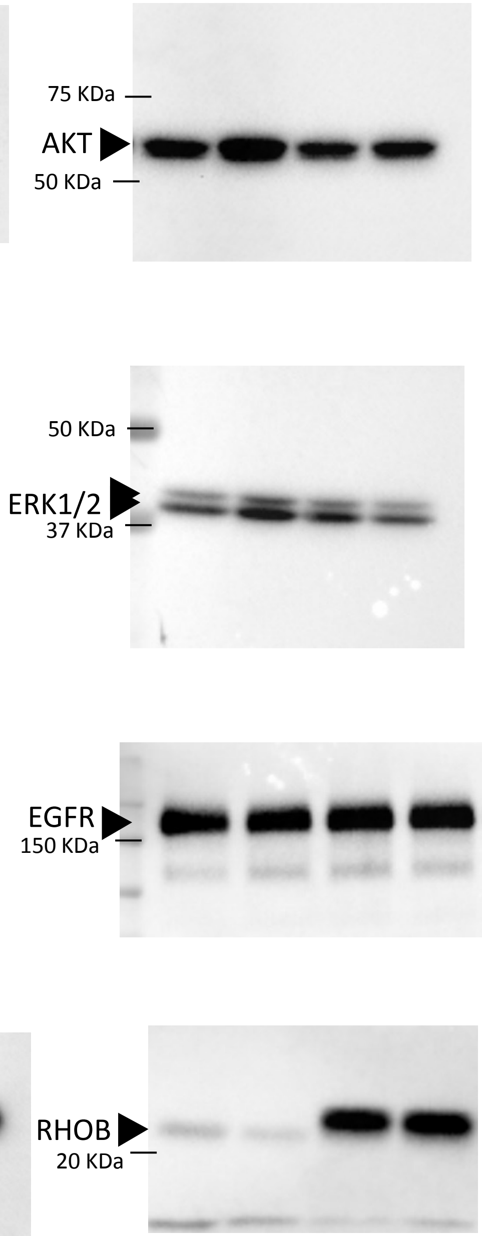

Fig. 4C

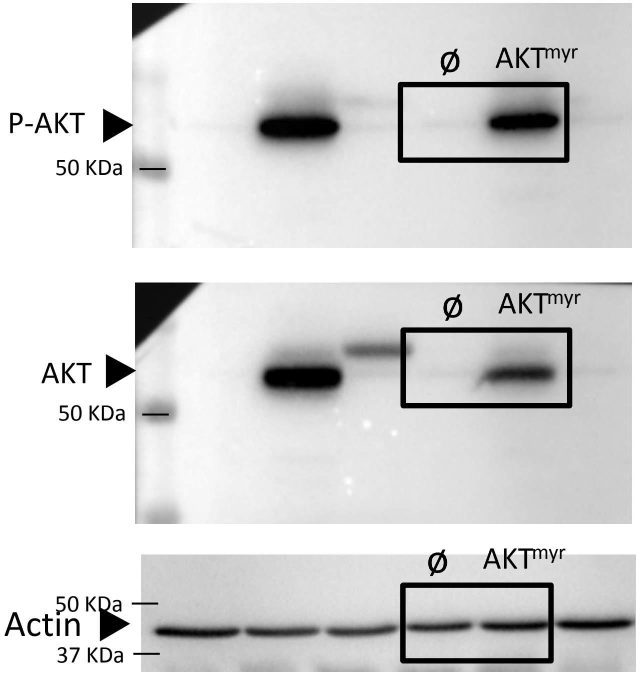

Ø
AKT^myr
P-AKT
50 KDa
Ø
AKT^myr
AKT
50 KDa
Ø
AKT^myr
50 KDa
Actin
37 KDa
